# Supplementary material for: Disruptions to the procurement of medical abortion medicines during COVID-19: a scoping review
Source: BMJ Open. 2022 Oct 27;12(10):e064848. doi: 10.1136/bmjopen-2022-064848 (PMC9621154; doi:10.1136/bmjopen-2022-064848)
Supplement: Supplementary data [file bmjopen-2022-064848supp002.pdf]

Appendix 2: Inclusion/Exclusion and Search Strategy

	Inclusion	Exclusion
**Medical Abortion**	• Medical Abortion<br>• Misoprostol, mifepristone, combipack and/or oxytocin	• Abortion services<br>• Contraception<br>• Reproductive health or family planning supplies without mentioning abortion medications
**Covid-19**	• All terms related to the SARS-COV-2 pandemic	• Previous SARS outbreaks<br>• Other pandemics or epidemics
**Procurement**	• Characteristics of ordering and/or receiving medical abortion medicines<br>• Examples of adapting to medical abortion medicine supply disruption<br>• Characteristics or examples of medical abortion supply chain disruption affecting procurement	• Procurement of commodities not related to medical abortion
**Evidence base**	• Primary data provided<br>• Methodological basis for study	• Opinion or commentary<br>• Speculative content

Search strategy

**Type of studies**	All; no protocols
**Condition**	Unwanted pregnancy
**Time frame**	January 1, 2020 to April 12, 2022
**Type of publications**	Published and unpublished articles
**Language**	English
**Setting**	All settings

WHO COVID Database

Retrieved April 14, 2022

#	Searches
1. Medical Abortion	tw:"medical abortion" OR tw:"medication abortion" OR mh:misoprostol or mh:mifepristone OR tw:misoprostol OR tw:mifepristone OR mh:"postcoital contraception" OR tw:"postcoital contraception" OR tw:"emergency contraception" OR tw:"abortifacient agents" OR tw:mifegyne OR tw:mifégyne OR tw:mifeprex OR tw:cytotec OR tw:glefos OR tw:amethopterin OR tw:cityl OR tw:cyprostol OR tw:cytolog OR tw:gastotec OR tw:gastrul OR tw:gymiso OR tw:hemoprostol OR tw:isprelor OR tw:misel OR tw:misodel OR tw:misopress OR tw:misoprostil)
2. Covid-19	tw:"corona virus" OR " tw:corona pandemic" OR tw:coronavir* OR tw:"sars virus" OR tw:COVID OR tw:Covid-19 OR tw:pandemic
#1 AND #2 New Articles from WHO Database	380

Pubmed

Retrieved April 14, 2022

#	Searches
1. Medical Abortion	"Contraceptives, Postcoital"[MeSH] OR "medical abortion"[tw] OR "medication abortion"[tw] OR "Abortifacient Agents" [MeSH] OR Misoprostol[MeSH] OR "Mifepristone"[MeSH] OR Mifegyne[TW] OR Mifégyne[TW] OR Mifeprex[TW] OR Mifepristone[TW] OR amethopterin[TW] OR cityl[TW] OR cyprostol[TW] OR cytolog[TW] OR gastotec[TW] OR gastrul[TW] OR gymiso[TW] OR hemoprostol[TW] OR isprel[TW] OR misel[TW] OR misodel[TW] OR misopress[TW] OR misoprostil[TW] OR misotrol[TW] OR misoclear[TW] OR mysodelle[tw] OR "R 38486"[tw] OR R38486[tw] OR "RU 38486"[tw] OR "RU 486"[tw] OR RU38486 [tw] OR RU486[tw] OR RU-486[tw] OR ZK 98296 [tw] OR ZK98296 [TW] OR "misoprostol"[MeSH Terms] OR misoprostol[tw] OR Cytotec[TW] OR Glefos[TW] OR "SC 29333"[TW] OR "SC 30249"[TW] OR SC29333[TW] OR SC30249 [TW] OR Amethopterin [ALL] OR Mexateè[ALL] OR (("Prostaglandins E, Synthetic"[MeSH] OR "methotrexate"[MeSH]) AND ("abortion, induced"[MeSH] OR "Abortion, Criminal"[MeSH] OR "Abortion, Septic"[MeSH] OR "Abortion Applicants"[MeSH] OR "abortion, missed"[MeSH] OR "abortion, legal"[MeSH])) OR ("family planning"[tw] AND (suppl*[TW] OR commodity[TW] OR drugs[TW] OR products[TW]))
2.	"corona virus"[tiab] OR "corona pandemic"[tiab] OR coronavir*[tiab] OR betacoronavir*[tiab] OR covid19[tiab] OR covid*[tiab] OR "wuhan

Covid-19	virus"[tiab] OR "severe acute respiratory syndrome"[tiab] OR "novel CoV"[tiab] OR nCoV*[tiab] OR hcov[tiab] OR "CoV 2"[tiab] OR CoV2[tiab] OR 2019nCoV[tiab] OR NCOV19[tiab] OR ncovid[tiab] OR sarscov2[tiab] OR sars2[tiab] OR "sars 2"[tiab] OR "sars-cov"[tiab] OR "sarscov 2"[tiab] OR "sars cov 2"[tiab] OR "sars co v 2"[tiab] OR sarscov[tiab] OR "sars virus"[tiab] OR COVID-19[MeSH]
#1 AND #2 New Articles from PubMed	278

Science Direct

Retrieved April 14, 2022

#	Searches
1. Medical Abortion (limit of 8 boolean operations/search)	1)"medical abortion"[tw] OR "medication abortion"[tw] OR misoprostol[tw] OR mifepristone[tw] OR "postcoital contraception"[tw] OR "emergency contraception"[tw] OR "abortifacient agents"[tw]<br><br>2)mifegyne[TW] OR mifégyne[TW] OR mifeprex[TW] OR cytotec [TW] OR glefos[TW] OR amethopterin[TW] OR cityl[TW] OR cyprostol[tw]<br><br>3)cytolog OR gastotec OR gastrul OR gymiso OR hemoprostol OR isprelor OR misel OR misodel<br><br>4) misopress OR misoprostil OR misotrol OR misoclear OR mysodelle OR R38486 OR ”RU 38486”<br><br>5) ”RU 486” OR RU38486 OR RU486 OR RU-486 OR ”ZK 98296” OR ZK98296 OR (”family planning” AND (suppl* OR commodity OR drugs OR products))
2. Covid-19	Covid-19
#1 AND #2 New Articles from Science Direct	135

PMC (Pubmed Central)

Retrieved April 14, 2022

#	Searches
1. Medical Abortion	"Contraceptives, Postcoital"[MeSH] OR "medical abortion"[tw] OR "medication abortion"[tw] OR "Abortifacient Agents" [MeSH] OR Misoprostol[MeSH] OR misoprostol[tw] OR "Mifepristone"[MeSH] OR

	Mifegyne[tw] OR Mifégyne[tw] OR Mifeprex[tw] OR Mifepristone[tw] OR amethopterin[tw] OR cityl[tw] OR cyprostol[tw] OR cytolog[tw] OR gastotec[tw] OR gastrul[tw] OR gymiso[tw] OR hemoprostol[tw] OR isprel[tw] OR misel[tw] OR misodel[tw] OR misopress[tw] OR misoprostil[tw] OR misotrol[tw] OR misoclear[tw] OR mysodelle[tw]OR Cytotec[tw] OR Glefos[tw])
2.<br><br>Covid-19	"corona virus"[tw] OR "corona pandemic"[tw] OR coronavir*[tw] OR betacoronavir*[tw] OR covid19[tw] OR covid*[tw] OR "wuhan virus"[tw] OR "severe acute respiratory syndrome"[tw] OR "novel CoV"[tw] OR nCoV*[tw] OR hcov[tw] OR "CoV 2"[tw] OR CoV2[tw] OR 2019nCoV[tw] OR NCOV19[tw] OR ncovid[tw] OR sarscov2[tw] OR sars2[tw] OR "sars 2"[tw] OR "sars-cov"[tw] OR "sarscov 2"[tw] OR "sars cov 2"[tw] OR "sars co v 2"[tw] OR sarscov[tw] OR "sars virus"[tw] OR COVID-19[MeSH]
#1 AND #2 New Articles from PMC	501

Cochrane Library

Retrieved April 14, 2022

#	Searches
1. Medical Abortion	#1MeSH descriptor: [Abortion, Induced] explode all trees<br><br>#2("medical abortion" OR "medication abortion" or mifepristone OR misoprostol OR ”postcoital contraception” OR ”emergency contraception” OR "abortifacient agents" OR mifegyne OR mifégyne OR mifeprex OR cytotec OR glefos):ti,ab,kw (Word variations have been searched)<br><br>#3”family planning” AND (suppl* OR commodity OR drugs OR products)
2. Covid-19	#4coronavirus 2019 OR covid OR coronavir* OR betacoronavir* OR covid19 OR covid* OR “wuhan virus” OR “severe acute respiratory syndrome” OR “novel CoV” OR nCoV* OR hcov OR CoV 2 OR CoV2 OR 2019nCoV OR NCOV19 OR sarscov2 OR sars2 OR sars 2 OR sars-cov OR “sarscov 2” OR “sars cov 2” OR “sars co v 2” OR sarscov OR “sars virus” OR COVID OR Covid-19 OR pandemic<br><br>#5MeSH descriptor: [SARS-CoV-2] explode all trees
(#1 OR #2 OR #3) AND (#4 AND #5) New Articles from the Cochrane Library:	8 [Removed 7 protocols)

EMBASE

Retrieved April 14, 2022

#	Searches
1. Medical Abortion	('contraceptives, postcoital':ti,ab,kw OR 'medical abortion':ti,ab,kw OR 'medication abortion':ti,ab,kw OR 'abortifacient agents':ti,ab,kw OR 'mifepristone':ti,ab,kw OR mifegyne:ti,ab,kw OR mifégyne:ti,ab,kw OR mifeprex:ti,ab,kw OR mifepristone:ti,ab,kw OR amethopterin:ti,ab,kw OR cityl:ti,ab,kw OR cyprostol:ti,ab,kw OR cytolog:ti,ab,kw OR gastotec:ti,ab,kw OR gastrul:ti,ab,kw OR gymiso:ti,ab,kw OR hemoprostol:ti,ab,kw OR isprel:ti,ab,kw OR misel:ti,ab,kw OR misodel:ti,ab,kw OR misopress:ti,ab,kw OR misoprostil:ti,ab,kw OR misotrol:ti,ab,kw OR misoclear:ti,ab,kw OR misoprostol:ti,ab,kw)
2. Covid-19	'coronavirus disease 2019'
#1 AND #2 New Articles from EMBASE	71
