# Supplementary material for: Disruptions to the procurement of medical abortion medicines during COVID-19: a scoping review
Source: BMJ Open. 2022 Oct 27;12(10):e064848. doi: 10.1136/bmjopen-2022-064848 (PMC9621154; doi:10.1136/bmjopen-2022-064848)
Supplement: Author's manuscript [file bmjopen-2022-064848.draft_revisions.pdf]

BMJ Open

BMJ Open is committed to open peer review. As part of this commitment we make the peer review history of every article we publish publicly available.

When an article is published we post the peer reviewers' comments and the authors' responses online. We also post the versions of the paper that were used during peer review. These are the versions that the peer review comments apply to.

The versions of the paper that follow are the versions that were submitted during the peer review process. They are not the versions of record or the final published versions. They should not be cited or distributed as the published version of this manuscript.

BMJ Open is an open access journal and the full, final, typeset and author-corrected version of record of the manuscript is available on our site with no access controls, subscription charges or pay-per-view fees (http://bmjopen.bmj.com).

If you have any questions on BMJ Open's open peer review process please email info.bmjopen@bmj.com

BMJ Open

Disruptions to the procurement of medical abortion medicines during COVID-19: a scoping review

Journal:	*BMJ Open*
Manuscript ID	bmjopen-2022-064848
Article Type:	Original research
Date Submitted by the Author:	17-May-2022

Complete List of Authors:	Cassinath, Natasha ; World Health Organization, Sexual and Reproductive Health and Research and UNDP-UNFPA-UNICEF-WHO-World Bank Special Programme of Research, Development and Research Training in Human Reproduction (HRP),<br>Titulaer, Patricia; World Health Organization, Sexual and Reproductive Health and Research and UNDP-UNFPA-UNICEF-WHO-World Bank Special Programme of Research, Development and Research Training in Human Reproduction (HRP),<br>Lavelaneta, Antonella ; World Health Organization, Department of Sexual and Reproductive Health and Research and UNDP-UNFPA-UNICEF-WHO-World Bank Special Programme of Research, Development and Research Training in Human Reproduction (HRP),<br>Läser, Laurence; World Health Organization, Sexual and Reproductive Health and Research and UNDP-UNFPA-UNICEF-WHO-World Bank Special Programme of Research, Development and Research Training in Human Reproduction (HRP),<br>Ahsan, Safia; Reproductive Health Supplies Coalition<br>Toedtli, Francelle ; United Nations Population Fund<br>Mawa , Stephen ; United Nations Population Fund<br>Rehnstrom Loi, Ulrika; World Health Organization, Sexual and Reproductive Health and Research and UNDP-UNFPA-UNICEF-WHO-World Bank Special Programme of Research, Development and Research Training in Human Reproduction (HRP),

Keywords:	COVID-19, PUBLIC HEALTH, Reproductive medicine < GYNAECOLOGY

SCHOLARONE™
Manuscripts

I, the Submitting Author has the right to grant and does grant on behalf of all authors of the Work (as defined in the below author licence), an exclusive licence and/or a non-exclusive licence for contributions from authors who are: i) UK Crown employees; ii) where BMJ has agreed a CC-BY licence shall apply, and/or iii) in accordance with the terms applicable for US Federal Government officers or employees acting as part of their official duties; on a worldwide, perpetual, irrevocable, royalty-free basis to BMJ Publishing Group Ltd ("BMJ") its licensees and where the relevant Journal is co-owned by BMJ to the co-owners of the Journal, to publish the Work in this journal and any other BMJ products and to exploit all rights, as set out in our licence.

The Submitting Author accepts and understands that any supply made under these terms is made by BMJ to the Submitting Author unless you are acting as an employee on behalf of your employer or a postgraduate student of an affiliated institution which is paying any applicable article publishing charge ("APC") for Open Access articles. Where the Submitting Author wishes to make the Work available on an Open Access basis (and intends to pay the relevant APC), the terms of reuse of such Open Access shall be governed by a Creative Commons licence – details of these licences and which Creative Commons licence will apply to this Work are set out in our licence referred to above.

Other than as permitted in any relevant BMJ Author's Self Archiving Policies, I confirm this Work has not been accepted for publication elsewhere, is not being considered for publication elsewhere and does not duplicate material already published. I confirm all authors consent to publication of this Work and authorise the granting of this licence.

For peer review only

Title

Disruptions to the procurement of medical abortion medicines during COVID-19: a scoping review

Authors

Natasha Cassinath[1], Patricia Titulaer[1], Laurence Läser[1], Antonella Lavelanet[1], Safia Ahsan[2], Francelle Kwankam Toedtli[3], Stephen Mawa[4], Ulrika Rehnström Loi[1]*

1. UNDP-UNFPA-UNICEF-WHO-World Bank Special Programme of Research, Development and Research Training in Human Reproduction (HRP), Department of Sexual and Reproductive Health and Research, World Health Organization, Geneva, Switzerland
2. Reproductive Health Supplies Coalition, Washington DC, USA
3. UNFPA, New York, New York, USA
4. UNFPA, Supply Chain Management Unit, Copenhagen, Denmark

*Corresponding author: Ulrika Rehnström Loi: rehnstromu@who.int

Word Count: 2943 (without abstract, boxes, tables and figures, references or appendices)

For peer review only

ABSTRACT

Objective
This scoping review aimed to systematically search, retrieve and map the extent and characteristics of available literature on the evidenced disruptions to medical abortion medicine procurement caused by the COVID-19 outbreak.

Methods
This scoping review considered studies related to abortion medicines and COVID-19 in PUBMED, EMBASE, PMC, Science Direct, the Cochrane Library, and Google Scholar from any country, in English, dated from January 2020 to April 2022.

Results
The two articles included are unpublished grey literature demonstrating evidence of short-lived disruptions in sexual and reproductive health commodity procurement, including medical abortion medicines, in Sub-Saharan Africa during the early months of the pandemic. Though limited, the findings indicate that preparedness, adaptations, and flexibility of key actors, including donors, largely addressed the disruptions.

Conclusion
The lack of evidence does not constitute proof that disruptions to medical abortion procurement were insignificant. There is a need for increased empirical evidence of medical abortion procurement challenges to understand which barriers to medical abortion procurement may persist and impact continuity of supply while others can fuel resilience and preparedness efforts at the country and sub-regional context. The role and resilience of social marketing organizations and their networks require a spotlight as they constitute a vital artery in the distribution of medical abortion commodities in low- and middle-income countries.

Keywords: procurement, medical abortion, COVID-19, supply chain, disruption, preparedness

For peer review only

BOX 1

What is already known on this topic

- Early in the pandemic, medical abortion stakeholders raised concerns about the timely availability and access to medical abortion products due to COVID-19 mitigation measures and related global and domestic supply chain issues.
- There were anecdotal reports of stockouts, fluctuating demand and other supply chain disruptions but no peer-reviewed research on the subject.

What this study adds

- The scoping review concludes that there is a dearth of evidence on the disruptions to medical abortion medicines procurement during the pandemic.
- In the limited contexts covered by this scoping review, there are signposts indicating the medical abortion supply chain has a built-in level of resilience to compensate for chronic risks.
- There is uncertainty with regards to the limits of this resilience. It may wear over several waves of COVID-19 given the anticipated increased costs of products and logistics, and a tighter financing landscape.

How this study might affect research, practice or policy

- Evidence gaps and future research highlighted include the need to 1) improve and increase the empirical evidence on MA medicines procurement and the greater supply chain, and 2) improve the resilience and preparedness of state and private health systems distributing MA medicines.
- We highlight the need to conduct research at national or subnational levels where market fragility is known to exist to support an expanded definition of risk mitigation and preparedness efforts.
- The contribution of social marketing organizations and their network merits comprehensive review as these actors distribute the bulk of medical abortion medicines in low- and middle-income countries worldwide and have outsized influence and experience in the procurement and distribution of medical abortion products during times of crisis and beyond.

For peer review only

INTRODUCTION

Medical Abortion (MA) medicines advanced the ability of women[1] to obtain abortion safely, at home, and with limited or no interactions with healthcare providers, if desired. MA medicines figure on the World Health Organization (WHO) list of essential medicines and are considered vital for comprehensive and post abortion care. When COVID-19 was declared a public health emergency of international concern by the WHO in January 2020, the restrictions in movement of goods and people put the procurement of MA medicines worldwide in jeopardy.

Procurement is the act of obtaining goods and services at an appropriate level of quality, at a specified time and location, and at a negotiated price.[1] It relies on established relationships with domestic and international suppliers; the capacity to accurately forecast demand; and the management of the supply pipeline to avoid stockouts, warehousing challenges or product expiry. All of these complex activities were reportedly compromised when domestic and international manufacturing, transport, donors, and regulations were impacted by COVID-19 containment efforts, just as MA procurement was needed more than ever.[2]

In some settings, MA medicines obtained by mail or locally with or without health provider assistance presented women with the option to bypass lockdown or distancing restrictions, and in others, accelerated the political acceptance of self-managed abortion as it became an urgent necessity for service continuity.[3,4,5] When the virus containment measures started to strain global supply chains and divert funds and energy to personal protective equipment (PPE), key stakeholders anticipated that the procurement of MA medicines was at risk and called for urgent action and preparedness, including stockpiling and close surveillance of the supply chains to offset potential procurement challenges.[4,6,7,8,9,10,11]

The UNDP-UNFPA-UNICEF-WHO-World Bank Special Programme of Research, Development and Research Training in Human Reproduction (HRP) conducted a pre-scoping survey to better understand the impact of COVID-19 on the ability to fund and procure MA medicines. The twenty-two (22) key stakeholders, including United Nations Population Fund (UNFPA), non-governmental organisations (NGOs) and social marketing organizations (SMOs), shared an array of supply chain funding and procurement challenges, as well as successful adaptations, but no clear root causes of disruptions, pervasive challenges or mitigation measures.

The rationale for this scoping review was to explore what emerging evidence on MA medicines procurement disruptions existed over the first two years of the pandemic and identify research gaps. Understanding the causes of medical abortion supply chain fragility or resilience in the short and longer term is essential to the development of mechanisms to forecast and plan for reliable supply in uncertain future scenarios. This understanding is of interest to comprehensive abortion care stakeholders, including actors involved in MA supply procurement, risk management, emergency preparedness, and donors. Thus, the aim of this scoping review is to systematically search, retrieve and map the extent and characteristics of available literature on the disruptions to MA medicine procurement during the COVID-19 outbreak.

---

[1] *Women* in this scoping review is intended to be construed inclusively to include girls and all persons who can become pregnant.

For peer review only

METHODS

Study design

We undertook a scoping review to explore the nature and extent of the evidence related to disruptions to procurement of MA medicines during COVID-19. We considered further areas of inquiry related to the types of disruption, causes and adaptations, but intentionally left the question broad to be as inclusive as possible. The methods for this review were informed by Arksey and O'Malley's methodology and Levac et al's methodological enhancement.[12,13]. This paper adheres to the Preferred Reporting Items for Systematic Reviews and Meta-Analyses (PRISMA) extension for scoping reviews.[14] The PRISMA checklist can be found in appendix 1.

Supplementary data
Appendix 1: PRISMA checklist

Patient and public involvement statement

As this was a review of existing literature, there was no need to engage with the public.

Search strategy

We piloted different search strategies including the concepts of "medical abortion," "procurement," "covid-19" with no geographical limitations. One reviewer (NC) supported by four advisory team members (PT, LL, AL, UR) harvested keywords from similar studies and trialled these in PubMed. We developed the final search strategy in consultation with the WHO medical librarian. We reduced the number of concepts to two: "medical abortion" and "covid-19"and included studies from January 2020 (right before the pandemic was officially declared) to April 2022 (4th- 6th wave depending on location). The comprehensive search strategy is presented in appendix 2. We conducted a database search in PubMed, PubMed Central, Embase, Science Direct, the Cochrane Library and the WHO Covid-19 database between November 4-6, 2021 and refreshed it on April 12, 2022. We searched Google Scholar with additional filters ("supply" OR "supply chain" OR "disruption") to manage the volume of results. Additional grey literature was obtained by searching target organizations' websites (e.g., UNFPA, WHO, MSI Reproductive Choices, DKT International, Reproductive Health Supplies Coalition). The reviewers used forward and backward citation searching of all articles in the full text review stage to find any additional studies fitting the inclusion criteria. All search results were stored using EndNote™ reference manager software and uploaded to Covidence® systematic review software.[15,16]

Supplementary data
Appendix 2 – Search Strategy

Study selection and data extraction process

Two reviewers (NC, PT) screened all titles and abstracts for inclusion independently. Studies that appeared relevant in a first instance were subjected to a full text review. We included articles that met the following inclusion criteria: (1) contained information on MA medicines; (2) included descriptions of procurement disruptions, including those with examples, characteristics and/or statistics; (3) documented events during the COVID-19 pandemic; and 4) articles presenting primary data. These criteria were developed in consult with all team members, based on the preliminary results of the search. We considered all English language documents from the timespan referred to above. A full description of the search terms, results, and inclusion/exclusion criteria is found in Appendix 2.

For peer review only

We developed a data extraction form in a standard Excel spreadsheet. The data points included document and study characteristics: author(s), title, publication year, journal, country/region, aim, nature of disruptions, root causes, adaptations, and author's conclusions. Two reviewers independently conducted the data abstraction. Each reviewer's extraction was compiled into a single excel spreadsheet in Microsoft Excel for validation.[17]

Data synthesis

Categorized data in the extraction table (Table 1 and 2) was analyzed across studies to identify emerging themes and subthemes related to the research question. Debriefing sessions were held with two members of the team to improve rigour and reduce researcher bias. Limitations to the heterogeneity of results were acknowledged. Study quality was not assessed as this is not a requirement for scoping reviews.[12]

REVIEW FINDINGS

Figure 1 shows the PRISMA flow chart. The search strategy returned 1662 results from the search of 6 databases, google scholar and web search. Of these, we excluded 811 records that did not meet the inclusion criteria, and 816 duplicate records. We conducted a full text review of 35 articles. Ultimately, we extracted information from 2 documents.

Characteristics of Included Articles and Data Extraction

Table 1 outlines the characteristics of the two studies included in the scoping review. Both articles present national level data on supply chain disruptions to sexual and reproductive health (SRH) or family planning commodities including MA medicines. Table 2 outlines the data extracted from the selected articles.

Table 1: Study characteristics

Author(s) Year	Article type	Journal	Data Collection	Region	Aim	Commodity/ Medicine Discussed
RHSC 2021 [18]	Rapid Assessment	Grey literature	53 interviews conducted globally Data collected March -October 2020*	Global, with additional quantitative data from Ethiopia, Zambia, and Kenya	To understand the disruptions to SRH supply chains during the pandemic and chart a roadmap for supply chain resiliency	General SRH including MA
Otieno et al 2021 [19]	Rapid Needs Assessment	Grey literature	Survey data from 17 African ministries of health/Data collected July 2020	17 Sub Saharan African Countries	To assess and document continuity of SRH services with a focus on safe abortion, post abortion care and family planning services during the COVID -19 pandemic	Family planning and MA

* This study included pre-pandemic data. Pre-pandemic and covid specific data were distinguishable.

Table 2: Data extraction - Disruption, Adaptation and Author's Conclusions

Author(s) Year	Types of Disruption	Adaptations	Authors' conclusions

RHSC 2021.[18]	Quarantine at ports/border closures; manufacturing stoppage; shortage of freight containers; increased product and freight costs; lack of air cargo/increased demand for sea freight; workforce reduction; reduced demand for MA; uncertainty due to changing restrictions; regulatory approvals and reauthorization of products delayed	Diversification of delivery channel; transition to sea freight; mapping and tracking adaptations; dispensing guidelines were changed; funder flexibility and responsiveness; Policy guidance from WHO and UN agencies helped overcome bottlenecks, particularly around the movement of health products and the inclusion of SRH within essential services.	Manufacturing, logistics and systems (including policies and procedures) were most affected. Data from six countries showed no unusual changes in stock levels. Higher initial stock levels mitigated delays. Within 2–4 months, most companies and organizations resumed operations with new constraints. Many health supply chains rely on material inputs from China and India, including active pharmaceutical ingredients. Transportation will continue to be challenging.
Otieno et al 2021.[19]	Limited public procurement; Funding shortfall; General stock delay	None related specifically to procurement	MA drugs available in health facilities in 13 (76%) countries. Where not available, reasons included: abortion forbidden, drugs not in health facilities but available in the pharmacies, controlled drugs not available for MA, available but for induction of labor and for post-partum haemorrhage, and abortion drugs not included on the essential medicines list. Madagascar had no abortion care due to lack of trained healthcare workers.

The findings from the included articles were organized into five areas related to MA medicines procurement during the COVID-19 pandemic: 1) pre-pandemic stockout mitigation strategies and their effects during the pandemic; 2) procurement adaptations at the international and local levels made in response to the pandemic; 3) the impact of manufacturing and transport related disruptions to procurement; 4) enabling or disabling national policies put in place during the pandemic; and 5) cost increases and changes to budget management due to the pandemic.

Both articles described stockouts of SRH medicines as a regular occurrence in many countries pre-pandemic.[18,19] In some countries, this risk was mitigated by maintaining a large stockpile of some of these medicines. During COVID-19, this protective overstock may have served to lessen the levels of stockouts in the short term. However, by the end of 2020, there was evidence of increased stockout in Zambia, Ethiopia, and Kenya, where stocks had otherwise remained stable.[18] In other Sub-Saharan Africa countries, COVID-19 related stockouts of SRH commodities began in March 2020 or later and continued to occur until at least October 2020. During this period, procurers were faced with increased costs related to PPE and pandemic response, commodity purchase prices, and transport; limited time to negotiate or perform price comparisons; and the additional effort to secure the transport and fulfil the purchase orders. These hurdles were followed by the implementation of adaptations allowing for abortion services to return to pre-pandemic levels within "weeks, not months".[18] Adaptations included attempting to identify alternative suppliers; adjusting to limited transport options; mapping and tracking orders; lobbying for funder flexibility and responsiveness; redistributing stock; and advocating SRH and/or MA supplies priority.[18] Diversifying the supplier base was a key challenge as manufacturers are largely geographically concentrated in Asia.[18]

At the onset of the pandemic, manufacturers stated that fluctuations in demand, anticipated supply chain disruptions, and panic buying challenged their ability to plan and produce SRH supplies in step with actual demand but they were able to rapidly return to pre-pandemic production levels once they adopted COVID-19 protocols in their work environment.[18]

Replenishment stock of SRH commodities through usual channels proved challenging, but in some instances there was evidence of local coordination and collaborative management of inventory between procurers, such as service providers and retailers, allowing for healthy stocks in one region to be

For peer review only

redeployed to another.[19] WhatsApp was used to improve communication and support the coordination of stock availability and redistribution.[18,19]

National policies related to the movement of select goods and workers played a part in stalling the ability of procurers to receive MA medicines. When abortion was not immediately deemed an essential service, the flow of MA medicines was deprioritized and prone to delay.[19] When MA was designated as an essential medicine, it positively impacted the ability to receive and distribute MA medicines nationally.[18,19]

Increases in transport costs and the diversions of funds to PPE were found to have direct impacts on SRH and MA budgets.[18,19] However, the burden of these costs was not always carried by the procurer – manufacturers, wholesalers and donors also incurred a share of these costs.[18] In the short term, donor flexibility; budgetary reallocation from PPE to SRH commodities or vice versa; and/or the ability to increase prices downstream in the supply chain allowed for continued procurement. The WHO African Region found that health ministries' financing for procurement was stable in 2020 as funding had been allocated and orders were already placed pre-pandemic.[19]

Concerns related to post-pandemic costs were raised in both articles.[18,19] Manufacturers claimed that improving risk mitigation efforts for the future, such as by maintaining larger stockpiles or developing relationships with a more diversified supplier base, would drive up their costs and affect product pricing.[18] Both articles highlighted that transport costs may continue to be impacted by backlogs, travel restrictions, storage constraints and limited freight options.[18,20]

DISCUSSION

Despite the anticipation of catastrophic impacts of COVID-19 on the supply of abortion medicines, this scoping review, employing a systematic comprehensive search strategy to gather all types of available evidence, found an almost negligeable number of articles (2) on the topic, both grey literature.

The limited evidence uncovered that preparedness — namely buffer stocks— and rapid adaptation to new conditions by multiple stakeholders (donors, policy makers, manufacturers, NGOs) were key ingredients in ensuring the continuous supply of SRH commodities, including MA medicines, to clients.

Concerns around COVID-19 disruptions on family planning were justified based on past experience with Ebola and other events, but ultimately the available evidence indicates that the scale of disturbance was not as detrimental as was initially envisioned, and/or service returned to pre-pandemic levels once lockdown periods concluded. [20,21]

It remains to be known how expansively resilience was experienced, where, and how long it can be expected to hold given that continued COVID-19 risk mitigation measures, pre-emptive stockpiling, limited transport options, shipping container shortages and rising petrol prices will increase the cost of doing business. National health budgets will be stretched as COVID-19 testing and vaccination efforts continue, and funds may be redirected to general population health and mental health due to neglect over the last two years.[22,23] In addition, organizations or companies reliant on international donations and/or unable to absorb these increased costs are anticipated to face a tight financing landscape, particularly due to the reduced levels of funding for safe abortion worldwide.[24]

For peer review only

It cannot be overstated that medical abortion commodity medicines are difficult to obtain in many parts of the world and that “returning to normal” does not imply a satisfactory status quo, particularly in restrictive jurisdictions or fragmented markets where data sets are unavailable.

Strengths and Limitations

The strengths associated with this scoping review is that it included both published and un-published literature and provides on overview of current evidence in the field of MA medicine procurement caused by the COVID-19 outbreak. However, there are several limitations to this review. The available evidence was limited and not published in peer reviewed journals. There is only a brief discussion of procurement in each article, in favour of a broader discussion of supply chain issues affecting either SRH supplies broadly or family planning supplies specifically. The studies refer to SRH including MA, which made applicability of some interventions or conclusions difficult to attribute to MA procurement specifically. Importantly, the evidence from field-based nongovernmental or social marketing organizations is very limited in these studies though these actors are a primary vehicle of MA services and products in low- and middle-income countries. Additionally, the articles reviewed were heavily weighted towards the supply chain issues of Sub-Saharan African countries and may not be relevant or representative of the reality in geographies.

CONCLUSION

This scoping review identified evidence gaps and future research needs to 1) improve and increase the empirical evidence on MA medicines procurement and the greater supply chain, and 2) improve the resilience and preparedness of state and private health systems distributing MA medicines. Given the general nature of current research looking at multiple countries across multiple sexual and reproductive health products, further examination should be undertaken in countries, where market fragility is known to exist, to support risk mitigation and preparedness efforts. The most important contribution to this work would be in capturing the resilience of SMOs and their network, who distribute the bulk of medical abortion medicines in low- and middle-income countries worldwide. These organizations have outsized influence and experience in the procurement and distribution of medical abortion products.

Acknowledgements

The authors would like to acknowledge the input of Kavita Kothari, World Health Organization Librarian, Geneva, Switzerland for her suggestions and support in refining the search strategy.

Contributorship statement

The scoping review was conceptualized by URL, PT, SA, FKT. NC and PT conducted the review, including assessing literature and data synthesis. NC prepared the manuscript with intellectual contributions from all authors that also approved the final manuscript for publication.

Competing interests

For peer review only

None declared.

Funding

This work was supported by the UNDP-UNFPA-UNICEF-WHO-World Bank Special Programme of Research, Development and Research Training in Human Reproduction (HRP), a cosponsored programme executed by the World Health Organization (WHO).

Data Sharing Statement

No additional data available.

Disclaimer

The views expressed in this article are those of the authors and do not necessarily represent the views of, and should not be attributed to, the World Health Organization, or the United Nations Population Fund.

Patient consent for publication

Not required

Ethics approval

An ethics statement is not applicable because this study was based exclusively on published literature.

Figure legend

Figure 1. PRISMA flow chart

For peer review only

References

1 Turner RW. Supply management and procurement: From the basics to best-in-class. *J. Ross Publishing*; 2011 Oct 10.

2 IPPF. The role of quality medical abortion during the coronavirus crisis. IPPF[Internet]. June 2020

3 Skuster P, Dhillon J and Li J. Easing of Regulatory Barriers to Telemedicine Abortion in Response to COVID-19. *Front Glob Womens Health.* 2021. doi: 10.3389/FGWH.2021.705611

4 Todd-Gher J and Shah P. Abortion in the context of COVID-19: a human rights imperative, *Sex Reprod Health Matters*. 2020; 28:1, doi: 10.1080/26410397.2020.1758394

5 Kumar M, Daly M, De Plecker E, et al. Now is the time: a call for increased access to contraception and safe abortion care during the COVID-19 pandemic *BMJ Global Health* 2020;5:e003175.

6 Purdy C. How will COVID-19 affect global access to contraceptives–and what can we do about it? DevEx [Internet]. 2020 Mar 11.

7 Casas X. Protecting Women's Reproductive Health During the Pandemic. Human Rights Watch [Internet]. 2020 May 21.

8 Weinberger M, Hayes B, White J, et al. Doing Things Differently: What It Would Take to Ensure Continued Access to Contraception During COVID-19. *Glob Health Sci Pract.* 2020;8(2):169-75.

9 UNFPA. New UNFPA projections predict calamitous impact on women's health as COVID-19 pandemic continues. UNFPA [Internet]. April 2020

10 Women's Refugee Council. Disruptions and Adaptations: The Effects of COVID-19 on Contraceptive Services across the Humanitarian-Development Nexus. Women's Refugee Council [Internet]. December 2020.

11 Samandari G, Kapp N, Hamon C, et al. Challenges in the abortion supply chain: a call to action for evaluation research. *Reprod Health*. 2021;18.

12 Arksey H, O'Malley L. Scoping studies: towards a methodological framework. *Int J Soc Res* Methodol. 2005;8(1):19–32.

13 Levac D, Colquhoun H, O'Brien KK. Scoping studies: advancing the methodology. *Implement Sci.* 2010;5(1):1-9.

14 Tricco AC, Lillie E, Zarin W, et al. PRISMA Extension for Scoping Reviews (PRISMA-ScR): checklist and explanation. *Ann Intern Med* 2018 Oct 2;169(7):467-473.

15 The EndNote Team. Endnote 20. Clarivate. 2013.

16 Covidence systematic review software, Veritas Health Innovation, Melbourne, Australia. Available at www.covidence.org

For peer review only

17 Microsoft Corporation. Microsoft Excel [Internet]. 2018. Available from: https://office.microsoft.com/excel

18 Reproductive Health Supplies Coalition. Building Resilient Sexual and Reproductive Health Supply Chains During Covid-19 and Beyond. RHSC [Internet]. April 2021.

19 Otieno GO, Ouedraogo L, Nkurunziza T, et al. Continuity of Essential Sexual and Reproductive Health Services During Covid-19 Pandemic in the Who African Region. WHO [Internet]. 2021.

20 Banke-Thomas, A., Yaya, S. Looking ahead in the COVID-19 pandemic: emerging lessons learned for sexual and reproductive health services in low- and middle-income countries. *Reprod Health* 18, 248 (2021). doi:10.1186/s12978-021-01307-4

21 Wood SN, Karp C, OlaOlorun F, et al. Need for and use of contraception by women before and during COVID-19 in four sub-Saharan African geographies: results from population-based national or regional cohort surveys. *The Lancet Global Health.* 2021 Jun 1;9(6):e793-801.

22 Findling MG, Blendon RJ, Benson JM. Delayed Care with Harmful Health Consequences—Reported Experiences from National Surveys During Coronavirus Disease 2019. *JAMA.* 2020;1(12):e201463. doi:10.1001/jamahealthforum.2020.1463

23 Cutler DM, Summers LH. The COVID-19 Pandemic and the $16 Trillion Virus. *JAMA.* 2020;324(15):1495–1496. doi:10.1001/jama.2020.19759

24 McVeigh K. UK accused of 'abandoning' women with cuts to aid. The Guardian [Internet]. April 2021.

For peer review only

Figure 1. PRISMA flow chart

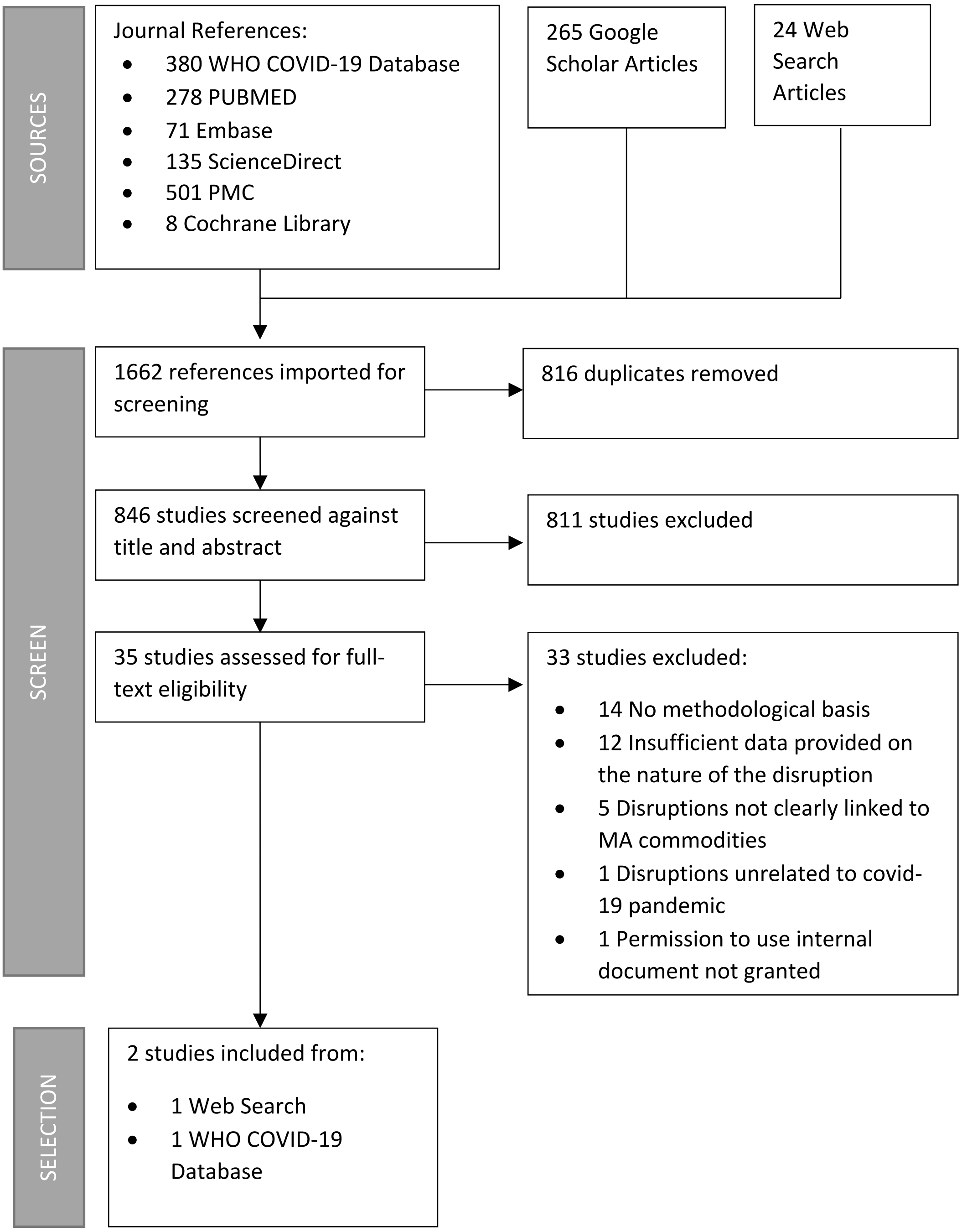

Appendix 1: Preferred Reporting Items for Systematic reviews and Meta-Analyses extension for Scoping Reviews (PRISMA-ScR) Checklist

SECTION	ITEM	PRISMA-ScR CHECKLIST ITEM	REPORTED ON PAGE #
**TITLE**			
Title	1	Identify the report as a scoping review.	1
**ABSTRACT**			
Structured summary	2	Provide a structured summary that includes (as applicable): background, objectives, eligibility criteria, sources of evidence, charting methods, results, and conclusions that relate to the review questions and objectives.	2
**INTRODUCTION**			
Rationale	3	Describe the rationale for the review in the context of what is already known. Explain why the review questions/objectives lend themselves to a scoping review approach.	4
Objectives	4	Provide an explicit statement of the questions and objectives being addressed with reference to their key elements (e.g., population or participants, concepts, and context) or other relevant key elements used to conceptualize the review questions and/or objectives.	3
**METHODS**			
Protocol and registration	5	Indicate whether a review protocol exists; state if and where it can be accessed (e.g., a Web address); and if available, provide registration information, including the registration number.	4
Eligibility criteria	6	Specify characteristics of the sources of evidence used as eligibility criteria (e.g., years considered, language, and publication status), and provide a rationale.	4
Information sources*	7	Describe all information sources in the search (e.g., databases with dates of coverage and contact with authors to identify additional sources), as well as the date the most recent search was executed.	4
Search	8	Present the full electronic search strategy for at least 1 database, including any limits used, such that it could be repeated.	Appendix 2
Selection of sources of evidence†	9	State the process for selecting sources of evidence (i.e., screening and eligibility) included in the scoping review.	5
Data charting process‡	10	Describe the methods of charting data from the included sources of evidence (e.g., calibrated forms or forms that have been tested by the team before their use, and whether data charting was done independently or in duplicate) and any processes for obtaining and confirming data from investigators.	5-6
Data items	11	List and define all variables for which data were sought and any assumptions and simplifications made.	5
Critical appraisal of individual sources of evidence§	12	If done, provide a rationale for conducting a critical appraisal of included sources of evidence; describe the methods used and how this information was used in any data synthesis (if appropriate).	n/a
Synthesis of results	13	Describe the methods of handling and summarizing the data that were charted.	5-6
**RESULTS**			

For peer review only

SECTION	ITEM	PRISMA-ScR CHECKLIST ITEM	REPORTED ON PAGE #
Selection of sources of evidence	14	Give numbers of sources of evidence screened, assessed for eligibility, and included in the review, with reasons for exclusions at each stage, ideally using a flow diagram.	Appendix 2
Characteristics of sources of evidence	15	For each source of evidence, present characteristics for which data were charted and provide the citations.	6, References
Critical appraisal within sources of evidence	16	If done, present data on critical appraisal of included sources of evidence (see item 12).	n/a
Results of individual sources of evidence	17	For each included source of evidence, present the relevant data that were charted that relate to the review questions and objectives.	6
Synthesis of results	18	Summarize and/or present the charting results as they relate to the review questions and objectives.	7-8
**DISCUSSION**			
Summary of evidence	19	Summarize the main results (including an overview of concepts, themes, and types of evidence available), link to the review questions and objectives, and consider the relevance to key groups.	8
Limitations	20	Discuss the limitations of the scoping review process.	9
Conclusions	21	Provide a general interpretation of the results with respect to the review questions and objectives, as well as potential implications and/or next steps.	10
**FUNDING**			
Funding	22	Describe sources of funding for the included sources of evidence, as well as sources of funding for the scoping review. Describe the role of the funders of the scoping review.	10

PRISMA-ScR = Preferred Reporting Items for Systematic reviews and Meta-Analyses extension for Scoping Reviews.

* Where *sources of evidence* (see second footnote) are compiled from, such as bibliographic databases, social media platforms, and Web sites.

† A more inclusive/heterogeneous term used to account for the different types of evidence or data sources (e.g., quantitative and/or qualitative research, expert opinion, and policy documents) that may be eligible in a scoping review as opposed to only studies. This is not to be confused with *information sources* (see first footnote).

‡ The frameworks by Arksey and O'Malley (6) and Levac and colleagues (7) and the JBI guidance (4, 5) refer to the process of data extraction in a scoping review as data charting.

§ The process of systematically examining research evidence to assess its validity, results, and relevance before using it to inform a decision. This term is used for items 12 and 19 instead of "risk of bias" (which is more applicable to systematic reviews of interventions) to include and acknowledge the various sources of evidence that may be used in a scoping review (e.g., quantitative and/or qualitative research, expert opinion, and policy document).

From: Tricco AC, Lillie E, Zarin W, O'Brien KK, Colquhoun H, Levac D, et al. PRISMA Extension for Scoping Reviews (PRISMAScR): Checklist and Explanation. Ann Intern Med. 2018;169:467–473. doi: 10.7326/M18-0850.

For peer review only - http://bmjopen.bmj.com/site/about/guidelines.xhtml

Appendix 2: Inclusion/Exclusion and Search Strategy

	Inclusion	Exclusion
**Medical Abortion**	• Medical Abortion<br>• Misoprostol, mifepristone, combipack and/or oxytocin	• Abortion services<br>• Contraception<br>• Reproductive health or family planning supplies without mentioning abortion medications
**Covid-19**	• All terms related to the SARS-COV-2 pandemic	• Previous SARS outbreaks<br>• Other pandemics or epidemics
**Procurement**	• Characteristics of ordering and/or receiving medical abortion medicines<br>• Examples of adapting to medical abortion medicine supply disruption<br>• Characteristics or examples of medical abortion supply chain disruption affecting procurement	• Procurement of commodities not related to medical abortion
**Evidence base**	• Primary data provided<br>• Methodological basis for study	• Opinion or commentary<br>• Speculative content

Search strategy

**Type of studies**	All; no protocols
**Condition**	Unwanted pregnancy
**Time frame**	January 1, 2020 to April 12, 2022
**Type of publications**	Published and unpublished articles
**Language**	English
**Setting**	All settings

For peer review only

WHO COVID Database

Retrieved April 14, 2022

#	Searches
1. Medical Abortion	tw:"medical abortion" OR tw:"medication abortion" OR mh:misoprostol or mh:mifepristone OR tw:misoprostol OR tw:mifepristone OR mh:"postcoital contraception" OR tw:"postcoital contraception" OR tw:"emergency contraception" OR tw:"abortifacient agents" OR tw:mifegyne OR tw:mifégyne OR tw:mifeprex OR tw:cytotec OR tw:glefos OR tw:amethopterin OR tw:cityl OR tw:cyprostol OR tw:cytolog OR tw:gastotec OR tw:gastrul OR tw:gymiso OR tw:hemoprostol OR tw:isprelor OR tw:misel OR tw:misodel OR tw:misopress OR tw:misoprostil)
2. Covid-19	tw:"corona virus" OR " tw:corona pandemic" OR tw:coronavir* OR tw:"sars virus" OR tw:COVID OR tw:Covid-19 OR tw:pandemic
#1 AND #2 New Articles from WHO Database	380

Pubmed

Retrieved April 14, 2022

#	Searches
1. Medical Abortion	"Contraceptives, Postcoital"[MeSH] OR "medical abortion"[tw] OR "medication abortion"[tw] OR "Abortifacient Agents" [MeSH] OR Misoprostol[MeSH] OR "Mifepristone"[MeSH] OR Mifegyne[TW] OR Mifégyne[TW] OR Mifeprex[TW] OR Mifepristone[TW] OR amethopterin[TW] OR cityl[TW] OR cyprostol[TW] OR cytolog[TW] OR gastotec[TW] OR gastrul[TW] OR gymiso[TW] OR hemoprostol[TW] OR isprel[TW] OR misel[TW] OR misodel[TW] OR misopress[TW] OR misoprostil[TW] OR misotrol[TW] OR misoclear[TW] OR mysodelle[tw] OR "R 38486"[tw] OR R38486[tw] OR "RU 38486"[tw] OR "RU 486"[tw] OR RU38486 [tw] OR RU486[tw] OR RU-486[tw] OR ZK 98296 [tw] OR ZK98296 [TW] OR "misoprostol"[MeSH Terms] OR misoprostol[tw] OR Cytotec[TW] OR Glefos[TW] OR "SC 29333"[TW] OR "SC 30249"[TW] OR SC29333[TW] OR SC30249 [TW] OR Amethopterin [ALL] OR Mexateè[ALL] OR (("Prostaglandins E, Synthetic"[MeSH] OR "methotrexate"[MeSH]) AND ("abortion, induced"[MeSH] OR "Abortion, Criminal"[MeSH] OR "Abortion, Septic"[MeSH] OR "Abortion Applicants"[MeSH] OR "abortion, missed"[MeSH] OR "abortion, legal"[MeSH])) OR ("family planning"[tw] AND (suppl*[TW] OR commodity[TW] OR drugs[TW] OR products[TW]))
2.	"corona virus"[tiab] OR "corona pandemic"[tiab] OR coronavir*[tiab] OR betacoronavir*[tiab] OR covid19[tiab] OR covid*[tiab] OR "wuhan

Covid-19	virus"[tiab] OR "severe acute respiratory syndrome"[tiab] OR "novel CoV"[tiab] OR nCoV*[tiab] OR hcov[tiab] OR "CoV 2"[tiab] OR CoV2[tiab] OR 2019nCoV[tiab] OR NCOV19[tiab] OR ncovid[tiab] OR sarscov2[tiab] OR sars2[tiab] OR "sars 2"[tiab] OR "sars-cov"[tiab] OR "sarscov 2"[tiab] OR "sars cov 2"[tiab] OR "sars co v 2"[tiab] OR sarscov[tiab] OR "sars virus"[tiab] OR COVID-19[MeSH]
#1 AND #2 New Articles from PubMed	278

Science Direct

Retrieved April 14, 2022

#	Searches
1. Medical Abortion (limit of 8 boolean operations/search)	1)"medical abortion"[tw] OR "medication abortion"[tw] OR misoprostol[tw] OR mifepristone[tw] OR "postcoital contraception"[tw] OR "emergency contraception"[tw] OR "abortifacient agents"[tw]<br><br>2)mifegyne[TW] OR mifégyne[TW] OR mifeprex[TW] OR cytotec [TW] OR glefos[TW] OR amethopterin[TW] OR cityl[TW] OR cyprostol[tw]<br><br>3)cytolog OR gastotec OR gastrul OR gymiso OR hemoprostol OR isprelor OR misel OR misodel<br><br>4) misopress OR misoprostil OR misotrol OR misoclear OR mysodelle OR R38486 OR ”RU 38486”<br><br>5) ”RU 486” OR RU38486 OR RU486 OR RU-486 OR ”ZK 98296” OR ZK98296 OR (”family planning” AND (suppl* OR commodity OR drugs OR products))
2. Covid-19	Covid-19
#1 AND #2 New Articles from Science Direct	135

PMC (Pubmed Central)

Retrieved April 14, 2022

#	Searches
1. Medical Abortion	"Contraceptives, Postcoital"[MeSH] OR "medical abortion"[tw] OR "medication abortion"[tw] OR "Abortifacient Agents" [MeSH] OR Misoprostol[MeSH] OR misoprostol[tw] OR "Mifepristone"[MeSH] OR

For peer review only

	Mifegyne[tw] OR Mifégyne[tw] OR Mifeprex[tw] OR Mifepristone[tw] OR amethopterin[tw] OR cityl[tw] OR cyprostol[tw] OR cytolog[tw] OR gastotec[tw] OR gastrul[tw] OR gymiso[tw] OR hemoprostol[tw] OR isprel[tw] OR misel[tw] OR misodel[tw] OR misopress[tw] OR misoprostil[tw] OR misotrol[tw] OR misoclear[tw] OR mysodelle[tw]OR Cytotec[tw] OR Glefos[tw])
2.<br><br>Covid-19	"corona virus"[tw] OR "corona pandemic"[tw] OR coronavir*[tw] OR betacoronavir*[tw] OR covid19[tw] OR covid*[tw] OR "wuhan virus"[tw] OR "severe acute respiratory syndrome"[tw] OR "novel CoV"[tw] OR nCoV*[tw] OR hcov[tw] OR "CoV 2"[tw] OR CoV2[tw] OR 2019nCoV[tw] OR NCOV19[tw] OR ncovid[tw] OR sarscov2[tw] OR sars2[tw] OR "sars 2"[tw] OR "sars-cov"[tw] OR "sarscov 2"[tw] OR "sars cov 2"[tw] OR "sars co v 2"[tw] OR sarscov[tw] OR "sars virus"[tw] OR COVID-19[MeSH]
#1 AND #2 New Articles from PMC	501

Cochrane Library

Retrieved April 14, 2022

#	Searches
1. Medical Abortion	#1MeSH descriptor: [Abortion, Induced] explode all trees<br><br>#2("medical abortion" OR "medication abortion" or mifepristone OR misoprostol OR ”postcoital contraception” OR ”emergency contraception” OR "abortifacient agents" OR mifegyne OR mifégyne OR mifeprex OR cytotec OR glefos):ti,ab,kw (Word variations have been searched)<br><br>#3”family planning” AND (suppl* OR commodity OR drugs OR products)
2. Covid-19	#4coronavirus 2019 OR covid OR coronavir* OR betacoronavir* OR covid19 OR covid* OR “wuhan virus” OR “severe acute respiratory syndrome” OR “novel CoV” OR nCoV* OR hcov OR CoV 2 OR CoV2 OR 2019nCoV OR NCOV19 OR sarscov2 OR sars2 OR sars 2 OR sars-cov OR “sarscov 2” OR “sars cov 2” OR “sars co v 2” OR sarscov OR “sars virus” OR COVID OR Covid-19 OR pandemic<br><br>#5MeSH descriptor: [SARS-CoV-2] explode all trees
(#1 OR #2 OR #3) AND (#4 AND #5) New Articles from the Cochrane Library:	8 [Removed 7 protocols)

For peer review only

EMBASE

Retrieved April 14, 2022

#	Searches
1. Medical Abortion	('contraceptives, postcoital':ti,ab,kw OR 'medical abortion':ti,ab,kw OR 'medication abortion':ti,ab,kw OR 'abortifacient agents':ti,ab,kw OR 'mifepristone':ti,ab,kw OR mifegyne:ti,ab,kw OR mifégyne:ti,ab,kw OR mifeprex:ti,ab,kw OR mifepristone:ti,ab,kw OR amethopterin:ti,ab,kw OR cityl:ti,ab,kw OR cyprostol:ti,ab,kw OR cytolog:ti,ab,kw OR gastotec:ti,ab,kw OR gastrul:ti,ab,kw OR gymiso:ti,ab,kw OR hemoprostol:ti,ab,kw OR isprel:ti,ab,kw OR misel:ti,ab,kw OR misodel:ti,ab,kw OR misopress:ti,ab,kw OR misoprostil:ti,ab,kw OR misotrol:ti,ab,kw OR misoclear:ti,ab,kw OR misoprostol:ti,ab,kw)
2. Covid-19	'coronavirus disease 2019'
#1 AND #2 New Articles from EMBASE	71

For peer review only

BMJ Open

Disruptions to the procurement of medical abortion medicines during COVID-19: a scoping review

Journal:	*BMJ Open*
Manuscript ID	bmjopen-2022-064848.R1
Article Type:	Original research
Date Submitted by the Author:	01-Sep-2022
Complete List of Authors:	Cassinath, Natasha ; World Health Organization, Sexual and Reproductive Health and Research and UNDP-UNFPA-UNICEF-WHO-World Bank Special Programme of Research, Development and Research Training in Human Reproduction (HRP),<br>TITULAER , Patricia ; World Health Organization, Sexual and Reproductive Health and Research and UNDP-UNFPA-UNICEF-WHO-World Bank Special Programme of Research, Development and Research Training in Human Reproduction (HRP),<br>Läser, Laurence; World Health Organization, Sexual and Reproductive Health and Research and UNDP-UNFPA-UNICEF-WHO-World Bank Special Programme of Research, Development and Research Training in Human Reproduction (HRP),<br>Lavelaneta, Antonella ; World Health Organization, Department of Sexual and Reproductive Health and Research and UNDP-UNFPA-UNICEF-WHO-World Bank Special Programme of Research, Development and Research Training in Human Reproduction (HRP),<br>Ahsan, Safia; Reproductive Health Supplies Coalition<br>Toedtli, Francelle ; United Nations Population Fund<br>Mawa , Stephen ; United Nations Population Fund<br>Rehnstrom Loi, Ulrika; World Health Organization, Sexual and Reproductive Health and Research and UNDP-UNFPA-UNICEF-WHO-World Bank Special Programme of Research, Development and Research Training in Human Reproduction (HRP),
**Primary Subject Heading**:	Reproductive medicine
Secondary Subject Heading:	Obstetrics and gynaecology, Qualitative research
Keywords:	COVID-19, PUBLIC HEALTH, Reproductive medicine < GYNAECOLOGY

SCHOLARONE™
Manuscripts

I, the Submitting Author has the right to grant and does grant on behalf of all authors of the Work (as defined in the below author licence), an exclusive licence and/or a non-exclusive licence for contributions from authors who are: i) UK Crown employees; ii) where BMJ has agreed a CC-BY licence shall apply, and/or iii) in accordance with the terms applicable for US Federal Government officers or employees acting as part of their official duties; on a worldwide, perpetual, irrevocable, royalty-free basis to BMJ Publishing Group Ltd ("BMJ") its licensees and where the relevant Journal is co-owned by BMJ to the co-owners of the Journal, to publish the Work in this journal and any other BMJ products and to exploit all rights, as set out in our licence.

The Submitting Author accepts and understands that any supply made under these terms is made by BMJ to the Submitting Author unless you are acting as an employee on behalf of your employer or a postgraduate student of an affiliated institution which is paying any applicable article publishing charge ("APC") for Open Access articles. Where the Submitting Author wishes to make the Work available on an Open Access basis (and intends to pay the relevant APC), the terms of reuse of such Open Access shall be governed by a Creative Commons licence – details of these licences and which Creative Commons licence will apply to this Work are set out in our licence referred to above.

Other than as permitted in any relevant BMJ Author's Self Archiving Policies, I confirm this Work has not been accepted for publication elsewhere, is not being considered for publication elsewhere and does not duplicate material already published. I confirm all authors consent to publication of this Work and authorise the granting of this licence.

For peer review only

authorsTitle

Disruptions to the procurement of medical abortion medicines during COVID-19: a scoping review

Authors

Natasha Cassinath[1], Patricia Titulaer[1], Laurence Läser[1], Antonella Lavelanet[1], Safia Ahsan[2], Francelle Kwankam Toedtli[3], Stephen Mawa[4], Ulrika Rehnström Loi[1]*

1. UNDP-UNFPA-UNICEF-WHO-World Bank Special Programme of Research, Development and Research Training in Human Reproduction (HRP), Department of Sexual and Reproductive Health and Research, World Health Organization, Geneva, Switzerland

2. Reproductive Health Supplies Coalition, Washington DC, USA

3. UNFPA, New York, New York, USA

4. UNFPA, Supply Chain Management Unit, Copenhagen, Denmark

*Corresponding author: Ulrika Rehnström Loi: rehnstromu@who.int

Word Count: 3506 (without abstract, boxes, tables and figures, references, or appendices)

For peer review only

For peer review only - http://bmjopen.bmj.com/site/about/guidelines.xhtml

ABSTRACT

Objective

This scoping review aimed to systematically search, retrieve, and map the extent and characteristics of available literature on the evidenced disruptions to medical abortion medicine procurement caused by the COVID-19 outbreak.

Methods

This scoping review considered all studies related to abortion medicines and COVID-19 in PUBMED, EMBASE, PMC, Science Direct, the Cochrane Library, and Google Scholar from any country, in English, dated from January 2020 to April 2022.

Findings

The two articles included are unpublished grey literature demonstrating evidence of short-lived disruptions in sexual and reproductive health commodity procurement, including MA medicines, in Sub-Saharan Africa during the early months of the pandemic. Findings show that in these Sub-Saharan contexts, emergency preparedness, stockpiling, adaptations, and flexibility of key actors, including donors, largely addressed the Covid-19 disruptions allowing for resumption of services within weeks.

Conclusion

The lack of evidence does not constitute proof that disruptions to medical abortion procurement were insignificant. There is a need for increased empirical evidence of medical abortion procurement challenges to understand which barriers to medical abortion procurement may persist and impact continuity of supply while others can fuel resilience and preparedness efforts at the country and sub-regional levels. The lack of evidence from social marketing organizations and their networks is a significant gap as these actors constitute a vital artery in the distribution of medical abortion commodities in low- and middle-income countries.

Keywords: procurement, medical abortion, COVID-19, supply chain, disruption, preparedness

For peer review only

Strengths and limitations of this study

- We considered 1,662 articles related to "medical abortion" and "covid-19".
- The study uncovered critical areas for future medical abortion supply chain research.
- The available evidence for extraction was limited to two articles which were not published in peer reviewed journals.
- The articles reviewed address supply chain issues of Sub-Saharan African countries with only a brief discussion of procurement in each article and unclear distinction of challenges by type of procurer.
- As studies refer to sexual and reproductive health including medical abortion, there is a risk that some interventions or conclusions are erroneously attributed to the challenge of medical abortion procurement.

For peer review only

For peer review only - http://bmjopen.bmj.com/site/about/guidelines.xhtml

INTRODUCTION

Globally, roughly 73.3 million abortions take place per year, at least half of which are performed through medical abortion.[1,2] Medical abortion (MA) is a procedure that terminates pregnancy using medicines - either mifepristone and misoprostol or misoprostol alone.[3] Mifepristone is a powerful abortifacient that blocks progesterone receptors and glucocorticoid receptors in the placenta to stop growth. Misoprostol dilates the cervix and induces muscle contractions which clears the uterus. MA is widely considered to be a safe and effective means to terminate pregnancy.

Medical Abortion (MA) medicines advanced the ability of women to obtain abortion safely, at home, and with limited or no interactions with healthcare providers, if desired. MA medicines figure on the World Health Organization (WHO) essential medicines list (EML) and are considered vital for comprehensive and post abortion care.[4,5] Country-based availability of MA medicines is restricted to where there is at least one legal ground for abortion. In 2019, 129 countries of a study of 158 had such a legal basis for abortion and, in principle, market authorization to stock and distribute MA medicines albeit in a controlled manner.[6]

Access to MA medicines is contingent on conducive policy, regulation, registration, EML inclusion, financing and supply chain management. When COVID-19 was declared a public health emergency of international concern by the WHO in January 2020, the restrictions in movement of goods and people strained supply chains and put the procurement of MA medicines in jeopardy. Procurement is the act of obtaining goods and services at an appropriate level of quality, at a specified time and location, and at a negotiated price.[7] It relies on established relationships with domestic and international suppliers; the capacity to accurately forecast demand; and the management of the supply pipeline to avoid stockouts, warehousing challenges or product expiry. All of these complex activities were reportedly compromised when domestic and international manufacturing, transport, donors, and regulations were impacted by COVID-19 containment efforts, just as MA procurement was needed more than ever.[8]

In some settings, MA medicines obtained by mail or locally with or without health provider assistance presented women with the option to bypass lockdown or distancing restrictions, and in others, accelerated the political acceptance of self-managed abortion as it became an urgent necessity for service continuity.[9,10,11] When the virus containment measures started to strain global supply chains and divert funds and energy to personal protective equipment (PPE), key stakeholders - armed with little information – anticipated that the procurement of MA

For peer review only - http://bmjopen.bmj.com/site/about/guidelines.xhtml

medicines was equally at risk and called for urgent action and preparedness, including stockpiling and close surveillance of the supply chains to offset potential procurement challenges.[12,13,14,15,16,17]

The UNDP-UNFPA-UNICEF-WHO-World Bank Special Programme of Research, Development and Research Training in Human Reproduction (HRP) conducted a pre-scoping survey to better understand the impact of COVID-19 on the ability to fund and procure MA medicines. There were twenty-two (22) respondents from United Nations Population Fund (UNFPA), non-governmental organisations (NGOs) and social marketing organizations . This resulted in a list of supply chain funding and procurement challenges, as well as successful adaptations. .However, no clear root causes of disruptions, pervasive challenges, or mitigation measures were identified. The rationale for this scoping review was to explore what emerging evidence on MA medicines procurement disruptions existed over the first two years of the pandemic and identify research gaps. Understanding the causes of MA supply chain fragility or resilience in the short and longer term is essential to the development of mechanisms to forecast and plan for reliable supply in uncertain future scenarios. This understanding is of interest to comprehensive abortion care stakeholders, including actors involved in MA supply procurement, risk management, emergency preparedness, and donors. Thus, the aim of this scoping review is to systematically search, retrieve and map the extent and characteristics of available literature on the disruptions to MA medicine procurement during the COVID-19 outbreak.

METHODS

Study design

We undertook a scoping review to explore the nature and extent of the evidence related to disruptions to procurement of MA medicines during COVID-19. We considered further areas of inquiry related to the types of disruption, causes and adaptations, but intentionally left the question broad to be as inclusive as possible. The methods for this review were informed by Arksey and O'Malley's methodology and Levac et al's methodological enhancement.[18,19]. This paper adheres to the Preferred Reporting Items for Systematic Reviews and Meta-Analyses (PRISMA) extension for scoping reviews.[20] The PRISMA checklist can be found in appendix 1.

Supplementary data

For peer review only - http://bmjopen.bmj.com/site/about/guidelines.xhtml

Appendix 1: PRISMA checklist

Patient and public involvement statement

During the planning phase we consulted partners and stakeholders through a virtual survey and several virtual meetings to engage them in the scoping review and support to make sense of the study findings.

Search strategy

We piloted different search strategies including the concepts of "medical abortion," "procurement," "covid-19" with no geographical limitations. One reviewer (NC) supported by four advisory team members (PT, LL, AL, UR) harvested keywords from similar studies and trialled these in PubMed. We developed the final search strategy in consultation with the WHO medical librarian. We reduced the number of concepts to two: "medical abortion" and "covid-19" and included studies from January 2020 (right before the pandemic was officially declared) to April 2022 (4th- 6th wave depending on location). The comprehensive search strategy is presented in appendix 2. We conducted a database search in PubMed, PubMed Central, Embase, Science Direct, the Cochrane Library and the WHO Covid-19 database between November 4-6, 2021 and refreshed it on April 12, 2022. We searched Google Scholar with additional filters ("supply" OR "supply chain" OR "disruption") to manage the volume of results. Additional grey literature was obtained by searching target organizations' websites (e.g., UNFPA, WHO, MSI Reproductive Choices, DKT International, Reproductive Health Supplies Coalition). The reviewers used forward and backward citation searching of all articles in the full text review stage to find any additional studies fitting the inclusion criteria. All search results were stored using EndNote™ reference manager software and uploaded to Covidence® systematic review software.[21,22]

Supplementary data

Appendix 2 – Search Strategy

Study selection and data extraction process

For peer review only - http://bmjopen.bmj.com/site/about/guidelines.xhtml

Two reviewers (NC, PT) screened all titles and abstracts for inclusion independently. Studies that appeared relevant in a first instance were subjected to a full text review. We included articles that met the following inclusion criteria: (1) contained information on MA medicines; (2) included descriptions of procurement disruptions, including those with examples, characteristics and/or statistics; (3) documented events during the COVID-19 pandemic; and 4) articles presenting primary data. These criteria were developed in consult with all team members, based on the preliminary results of the search. We considered all English language documents from the timespan referred to above. A full description of the search terms, results, and inclusion/exclusion criteria is found in Appendix 2.

We developed a data extraction form in a standard Excel spreadsheet. The data points included document and study characteristics: author(s), title, publication year, journal, country/region, aim, nature of disruptions, root causes, adaptations, and author's conclusions. Two reviewers independently conducted the data abstraction. Each reviewer's extraction was compiled into a single excel spreadsheet in Microsoft Excel for validation.[23]

Data synthesis

Categorized data in the extraction table (Table 1 and 2) was analyzed across studies to identify emerging themes and subthemes related to the research question. Debriefing sessions were held with two members of the team to improve rigour and reduce researcher bias. Limitations to the heterogeneity of results were acknowledged. Study quality was not assessed as this is not a requirement for scoping reviews.[18]

REVIEW FINDINGS

Figure 1 shows the PRISMA flow chart. The search strategy returned 1662 results from the search of 6 databases, google scholar and web search. Of these, we removed 816 duplicate records and excluded 811 records that did not meet the inclusion criteria. . We conducted a full text review of 35 articles. Ultimately, we extracted information from 2 documents.

For peer review only - http://bmjopen.bmj.com/site/about/guidelines.xhtml

Characteristics of Included Articles and Data Extraction

Table 1 outlines the characteristics of the two studies included in the scoping review. Both articles present national level data on supply chain disruptions to sexual and reproductive health (SRH) or family planning commodities including MA medicines. Table 2 outlines the data extracted from the selected articles.

Table 1: Study characteristics

Author(s) Year	Article type	Journal	Data Collection	Region	Aim	Commodity/ Medicine Discussed
RHSC 2021 [24]	Rapid Assessment	Grey literature	53 interviews conducted globally<br><br>Data collected March - October 2020*	Global, with additional quantitative data from Ethiopia, Zambia, and Kenya	To understand the disruptions to SRH supply chains during the pandemic and chart a roadmap for supply chain resiliency	General SRH including MA
Otieno et al 2021 [25]	Rapid Needs Assessment	Grey literature	Survey data from 17 African ministries of health/Data collected July 2020	17 Sub Saharan African Countries	To assess and document continuity of SRH services with a focus on safe abortion, post abortion care and family planning services during the COVID -19 pandemic	Family planning and MA

* This study included pre-pandemic data. Pre-pandemic and covid specific data were distinguishable.

Table 2: Data extraction - Disruption, Adaptation and Author’s Conclusions

For peer review only

For peer review only - http://bmjopen.bmj.com/site/about/guidelines.xhtml

Author(s) Year	Types of Disruption	Adaptations	Authors' conclusions
RHSC 2021.[24]	Quarantine at ports/border closures; manufacturing stoppage; shortage of freight containers; increased product and freight costs; lack of air cargo/increased demand for sea freight; workforce reduction; reduced demand for MA; uncertainty due to changing restrictions; regulatory approvals and delay in reauthorization of products	Diversification of delivery channel; transition to sea freight; mapping and tracking adaptations; dispensing guidelines were changed; funder flexibility and responsiveness; Policy guidance from WHO and UN agencies helped overcome bottlenecks, particularly around the movement of health products and the inclusion of SRH within essential services.	Manufacturing, logistics and systems (including policies and procedures) were most affected. Data from six countries showed no unusual changes in stock levels. Higher initial stock levels mitigated delays. Within 2–4 months, most companies and organizations resumed operations with new constraints. Many health supply chains rely on material inputs from China and India, including active pharmaceutical ingredients. Transportation will continue to be challenging.
Otieno et al 2021.[25]	Limited public procurement; Funding shortfall; General stock delay	None related specifically to procurement	MA drugs available in health facilities in 13 (76%) countries. Where not available, reasons included: abortion forbidden, drugs not in health facilities but available in the pharmacies, controlled drugs not available for MA, available but for induction of labour and for post-partum haemorrhage, and abortion drugs not included on the essential medicines list. Madagascar had no abortion care due to lack of trained healthcare workers.

The findings from the included articles were organized into five areas related to system-level MA medicines procurement during the COVID-19 pandemic: 1) pre-pandemic stockout mitigation strategies and their effects during the pandemic; 2) procurement adaptations at the international and local levels made in response to the pandemic; 3) the impact of manufacturing and transport related disruptions to procurement; 4) enabling or disabling national policies put in place during the pandemic; and 5) cost increases and changes to budget management due to the pandemic.

For peer review only - http://bmjopen.bmj.com/site/about/guidelines.xhtml

Both articles described stockouts of SRH medicines as a regular occurrence in many countries pre-pandemic.[24,25] In some countries, this risk was mitigated by maintaining a large stockpile of some of these medicines. During COVID-19, this protective overstock may have served to lessen the levels of stockouts in the short term. However, by the end of 2020, there was evidence of increased stockout in Zambia, Ethiopia, and Kenya, where stocks had otherwise remained stable.[24] In other Sub-Saharan Africa countries, COVID-19 related stockouts of SRH commodities began in March 2020 or later and continued to occur until at least October 2020. During this period, procurers were faced with increased costs related to PPE and pandemic response, commodity purchase prices, and transport; limited time to negotiate or perform price comparisons; and the additional effort to secure the transport and fulfil the purchase orders. These hurdles were followed by the implementation of adaptations allowing for abortion services to return to pre-pandemic levels within “weeks, not months”.[24] Adaptations included attempting to identify alternative suppliers; adjusting to limited transport options; mapping and tracking orders; lobbying for funder flexibility and responsiveness; redistributing stock; and advocating SRH and/or MA supplies priority.[24] Diversifying the supplier base was a key challenge as manufacturers are largely geographically concentrated in Asia.[24]

At the onset of the pandemic, manufacturers stated that fluctuations in demand, anticipated supply chain disruptions, and panic buying challenged their ability to plan and produce SRH supplies in step with actual demand but they were able to rapidly return to pre-pandemic production levels once they adopted COVID-19 protocols in their work environment.[24]

Replenishment stock of SRH commodities through usual channels proved challenging, but in some instances there was evidence of local coordination and collaborative management of inventory between procurers, such as service providers and retailers, allowing for healthy stocks in one region to be redeployed to another.[25] WhatsApp was used to improve communication and support the coordination of stock availability and redistribution.[24,25]

National policies related to the movement of select goods and workers played a part in stalling the ability of procurers to receive MA medicines. When abortion was not immediately deemed an essential service, the flow of MA medicines was deprioritized and prone to delay.[25] When MA was designated as an essential medicine, it positively impacted the ability to receive and distribute MA medicines nationally.[24,25]

For peer review only - http://bmjopen.bmj.com/site/about/guidelines.xhtml

Increases in transport costs and the diversions of funds to PPE were found to have direct impacts on SRH and MA budgets.[24,25] However, the burden of these costs was not always carried by the procurer – manufacturers, wholesalers and donors also incurred a share of these costs.[24] In the short term, donor flexibility; budgetary reallocation from PPE to SRH commodities or vice versa; and/or the ability to increase prices downstream in the supply chain allowed for continued procurement. The WHO African Region found that health ministries' financing for national procurement was stable in 2020 as funding had been allocated and orders were already placed pre-pandemic.[25]

Concerns related to post-pandemic costs were raised in both articles.[24,25] Manufacturers claimed that improving risk mitigation efforts for the future, such as by maintaining larger stockpiles or developing relationships with a more diversified supplier base, would drive up their costs and affect product pricing.[24] Both articles highlighted that transport costs may continue to be impacted by backlogs, travel restrictions, storage constraints and limited freight options.[24,25]

DISCUSSION

While there exists no straightforward data regarding the prevalence of medical abortion administration, proxy information from studies looking at the readiness of 210 health facilities in 11 African countries to provide post abortion care demonstrates that the primary challenge is not the availability of uterotonics (misoprostol or oxytocin), but rather, limitations of staff and critical care unit infrastructure.[26]

Before the COVID-19 pandemic, unintended pregnancy rates were higher in low- and middle-income countries (respectively, 93 and 66 unintended pregnancies per 1000 reproductive-aged women per year) than in high-income countries (34 per 1000).[1] Before the pandemic, women in low and middle income countries were also less likely to have a safe and legal abortion. For example, of 8.2 million annual abortions occuring annually in Africa, 76% are unsafe. Based on past experience of pandemics and other disruptive events, the magnitude of these disparities would be expected to increase the need and challenge the access to abortion services and products.[27]

Despite the anticipation of catastrophic impacts of COVID-19 on the supply of abortion medicines, this scoping review, employing a systematic comprehensive search strategy to gather all types of available evidence, found an almost negligeable number of articles (2) on the topic, both grey literature.

For peer review only - http://bmjopen.bmj.com/site/about/guidelines.xhtml

The limited evidence uncovered that preparedness — namely buffer stocks— and rapid adaptation to new conditions by multiple stakeholders (donors, policy makers, manufacturers, NGOs) were key ingredients in ensuring the continuous supply of SRH commodities, including MA medicines.

Concerns around COVID-19 disruptions on family planning were justified based on past experience, but ultimately the available evidence indicates that the scale of disturbance in the select Sub Saharan countries was not as detrimental as was initially envisioned, and/or service returned to pre-pandemic levels once lockdown periods concluded. [28,29]

It remains to be known how expansively resilience was experienced, where, and how long it can be expected to hold given that continued COVID-19 risk mitigation measures, pre-emptive stockpiling, limited transport options, shipping container shortages and rising petrol prices will increase the cost of doing business. National health budgets will be stretched as COVID-19 testing and vaccination efforts continue, and funds may be redirected to general population health and mental health due to neglect over the last two years.[30,31] In addition, organizations or companies reliant on international donations and/or unable to absorb these increased costs are anticipated to face a tight financing landscape, particularly due to the reduced levels of funding for safe abortion worldwide.[32]

It cannot be overstated that MA commodity medicines are difficult to obtain in many parts of the world and that “returning to normal” does not imply a satisfactory status quo, particularly in restrictive jurisdictions or fragmented markets where data sets are unavailable.

Strengths and Limitations

The strengths associated with this scoping review are that it includes both published and un-published literature and provides an overview of current evidence in the field of MA medicine procurement caused by the COVID-19 outbreak. Our focus was strictly on MA procurement and therefore, the availability of MA medicines does not infer access by clients. However, there are several limitations to this review. The available evidence was limited and not published in peer reviewed journals. There is only a brief discussion of procurement in each article, in favour of a broader discussion of supply chain issues affecting either SRH supplies broadly or family planning supplies specifically. The studies refer to SRH including MA, which made applicability of some interventions or conclusions difficult to attribute to MA procurement specifically.

For peer review only

Importantly, the evidence from field-based nongovernmental organizations and global social marketing organizations is very limited in these studies. Social marketing organizations are recognized as important actors in the procurement and distribution of MA services and products in low- and middle-income countries yet no published documentation of their experiences with MA procurement during the COVID-19 pandemic was found. Additionally, the articles reviewed were heavily weighted towards the supply chain issues of Sub-Saharan African countries and may not be relevant or representative of the reality in geographies.

CONCLUSION

This scoping review identified evidence gaps and future research needs to 1) improve and increase the empirical evidence on MA medicines procurement and the greater supply chain, and 2) improve the resilience and preparedness of state and private health systems distributing MA medicines. Given the general nature of current research looking at multiple countries across multiple sexual and reproductive health products, further examination should be undertaken in countries, where market fragility is known to exist, to support risk mitigation and preparedness efforts. The most important contribution to this work would be in capturing the resilience of social marketing organizations and their network, who distribute the bulk of MA medicines in low- and middle-income countries worldwide. These organizations have outsized influence and experience in the procurement and distribution of MA products, particularly in low and middle income countries.[33]

Acknowledgements

The authors would like to acknowledge the input of Kavita Kothari, World Health Organization Librarian, Geneva, Switzerland for her suggestions and support in refining the search strategy.

For peer review only

Contributorship statement

The scoping review was conceptualized by URL, PT, SA and FKT. NC and PT conducted the review, including assessing literature and data synthesis. NC prepared the first draft manuscript with significant intellectual contributions from PT, AL, LL, SA, FT, SM and URL. All authors that also reviewed and edited versions of the manuscript. The authors alone are responsible for the views expressed in this article, and they do not necessarily represent the views, decisions, or policies of the institutions with which they are affiliated. All authors approved the final manuscript for publication.

Competing interests

None declared.

Funding

This work was supported by the UNDP-UNFPA-UNICEF-WHO-World Bank Special Programme of Research, Development and Research Training in Human Reproduction (HRP), a cosponsored programme executed by the World Health Organization (WHO).

Data Sharing Statement

No additional data available.

For peer review only

Disclaimer

The views expressed in this article are those of the authors and do not necessarily represent the views of, and should not be attributed to, the World Health Organization, or the United Nations Population Fund.

Patient consent for publication

Not required

Ethics approval

An ethics statement is not applicable because this study was based exclusively on published literature.

Figure legend

Figure 1. PRISMA flow chart

For peer review only

For peer review only - http://bmjopen.bmj.com/site/about/guidelines.xhtml

References

1 Bearak J, Popinchalk A, Ganatra B, et al. Unintended pregnancy and abortion by income, region, and the legal status of abortion: estimates from a comprehensive model for 1990–2019. Lancet Glob Health 2020. doi: 10.1016/S2214-109X(20)30315-6.

2 Bankole A, Remez L, Owolabiet O, al., From Unsafe to Safe Abortion in Sub-Saharan Africa: Slow but Steady Progress, New York: Guttmacher Institute, 2020, https://www.guttmacher.org/report/ from-unsafe-to-safe-abortion-in-subsaharan-africa. doi:10.1363/2020.32446

3 Aronson JK, editor. Meyler's Side Effects of Drugs, Sixteenth Editions. 2016.

4 WHO. World Health Organization Model List of Essential Medicines – 22nd List, 2021. World Health Organization[Internet]. 2021.

5 WHO. Abortion care guideline. World Health Organization[Internet]. 2022.

6 Lavelanet, A.F., Schlitt, S., Johnson *et al.* Global Abortion Policies Database: a descriptive analysis of the legal categories of lawful abortion. *BMC Int Health Hum Rights* 18, 44 2018. doi.org/10.1186/s12914-018-0183-1

7 Turner RW. Supply management and procurement: From the basics to best-in-class. *J. Ross Publishing*; 2011 Oct 10.

8 IPPF. The role of quality medical abortion during the coronavirus crisis. IPPF[Internet]. June 2020

9 Skuster P, Dhillon J and Li J. Easing of Regulatory Barriers to Telemedicine Abortion in Response to COVID-19. *Front Glob Womens Health.* 2021. doi: 10.3389/FGWH.2021.705611

For peer review only - http://bmjopen.bmj.com/site/about/guidelines.xhtml

10 Todd-Gher J and Shah P. Abortion in the context of COVID-19: a human rights imperative, *Sex Reprod Health Matters*. 2020; 28:1, doi: 10.1080/26410397.2020.1758394

11 Kumar M, Daly M, De Plecker E, et al. Now is the time: a call for increased access to contraception and safe abortion care during the COVID-19 pandemic *BMJ Global Health* 2020;5:e003175.

12 Purdy C. How will COVID-19 affect global access to contraceptives–and what can we do about it? DevEx [Internet]. 2020 Mar 11.

13 Casas X. Protecting Women's Reproductive Health During the Pandemic. Human Rights Watch [Internet]. 2020 May 21.

14 Weinberger M, Hayes B, White J, et al. Doing Things Differently: What It Would Take to Ensure Continued Access to Contraception During COVID-19. *Glob Health Sci Pract.* 2020;8(2):169-75.

15 UNFPA. New UNFPA projections predict calamitous impact on women's health as COVID-19 pandemic continues. UNFPA [Internet]. April 2020

16 Women's Refugee Council. Disruptions and Adaptations: The Effects of COVID-19 on Contraceptive Services across the Humanitarian-Development Nexus. Women's Refugee Council [Internet]. December 2020.

17 Samandari G, Kapp N, Hamon C, et al. Challenges in the abortion supply chain: a call to action for evaluation research. *Reprod Health*. 2021;18.

18 Arksey H, O'Malley L. Scoping studies: towards a methodological framework. *Int J Soc Res* Methodol. 2005;8(1):19–32.

For peer review only

For peer review only - http://bmjopen.bmj.com/site/about/guidelines.xhtml

19 Levac D, Colquhoun H, O'Brien KK. Scoping studies: advancing the methodology. *Implement Sci.* 2010;5(1):1-9.

20 Tricco AC, Lillie E, Zarin W, et al. PRISMA Extension for Scoping Reviews (PRISMA-ScR): checklist and explanation. *Ann Intern Med* 2018 Oct 2;169(7):467-473.

21 The EndNote Team. Endnote 20. Clarivate. 2013.

22 Covidence systematic review software, Veritas Health Innovation, Melbourne, Australia. Available at www.covidence.org

23 Microsoft Corporation. Microsoft Excel [Internet]. 2018. Available from: https://office.microsoft.com/excel

24 Reproductive Health Supplies Coalition. Building Resilient Sexual and Reproductive Health Supply Chains During Covid-19 and Beyond. RHSC [Internet]. April 2021.

25 Otieno GO, Ouedraogo L, Nkurunziza T, et al. Continuity of Essential Sexual and Reproductive Health Services During Covid-19 Pandemic in the Who African Region. WHO [Internet]. 2021.

26 Compaoré, R, Mehrtash, H, Calvert, C, et al. Health facilities' capability to provide comprehensive postabortion care in Sub-Saharan Africa: Evidence from a cross-sectional survey across 210 high-volume facilities. Int J Gynecol Obstet. 2022; 156(Suppl. 1): 7– 19. doi:10.1002/ijgo.14056

27 Wenham C, Smith J, Davies SE, et al. Women are most affected by pandemics- lessons from past outbreaks. Nature. 2020. doi: 10.1038/d41586-020-02006-z

For peer review only

For peer review only - http://bmjopen.bmj.com/site/about/guidelines.xhtml

28 Banke-Thomas, A., Yaya, S. Looking ahead in the COVID-19 pandemic: emerging lessons learned for sexual and reproductive health services in low- and middle-income countries. Reprod Health.2021. doi:10.1186/s12978-021-01307-4

29 Wood SN, Karp C, OlaOlorun F, et al. Need for and use of contraception by women before and during COVID-19 in four sub-Saharan African geographies: results from population-based national or regional cohort surveys. *The Lancet Global Health.* 2021 Jun 1;9(6):e793-801.

30 Findling MG, Blendon RJ, Benson JM. Delayed Care with Harmful Health Consequences—Reported Experiences from National Surveys During Coronavirus Disease 2019. *JAMA.* 2020;1(12):e201463. doi:10.1001/jamahealthforum.2020.1463

31 Cutler DM, Summers LH. The COVID-19 Pandemic and the $16 Trillion Virus. *JAMA.* 2020;324(15):1495–1496. doi:10.1001/jama.2020.19759

32 McVeigh K. UK accused of 'abandoning' women with cuts to aid. The Guardian [Internet]. April 2021.

33 Mann Global Health. Landscape assessment: leveraging the role of national distributors to increase access to MA combi-packs in Africa [Internet]. 2019.

For peer review only

For peer review only - http://bmjopen.bmj.com/site/about/guidelines.xhtml

Figure 1. PRISMA flow chart

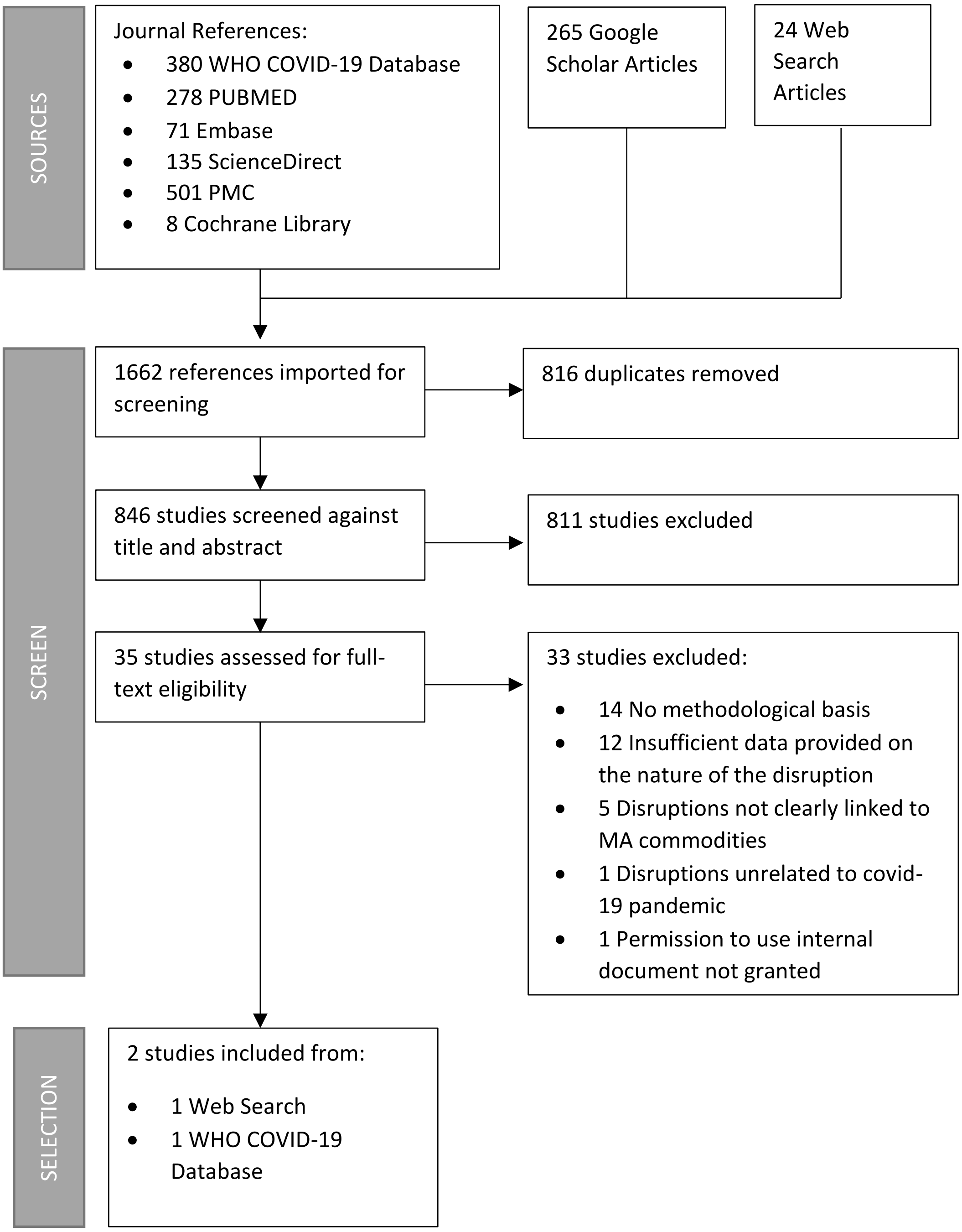

Appendix 1: Preferred Reporting Items for Systematic reviews and Meta-Analyses extension for Scoping Reviews (PRISMA-ScR) Checklist

SECTION	ITEM	PRISMA-ScR CHECKLIST ITEM	REPORTED ON PAGE #
**TITLE**			
Title	1	Identify the report as a scoping review.	1
**ABSTRACT**			
Structured summary	2	Provide a structured summary that includes (as applicable): background, objectives, eligibility criteria, sources of evidence, charting methods, results, and conclusions that relate to the review questions and objectives.	2
**INTRODUCTION**			
Rationale	3	Describe the rationale for the review in the context of what is already known. Explain why the review questions/objectives lend themselves to a scoping review approach.	4
Objectives	4	Provide an explicit statement of the questions and objectives being addressed with reference to their key elements (e.g., population or participants, concepts, and context) or other relevant key elements used to conceptualize the review questions and/or objectives.	3
**METHODS**			
Protocol and registration	5	Indicate whether a review protocol exists; state if and where it can be accessed (e.g., a Web address); and if available, provide registration information, including the registration number.	4
Eligibility criteria	6	Specify characteristics of the sources of evidence used as eligibility criteria (e.g., years considered, language, and publication status), and provide a rationale.	4
Information sources*	7	Describe all information sources in the search (e.g., databases with dates of coverage and contact with authors to identify additional sources), as well as the date the most recent search was executed.	4
Search	8	Present the full electronic search strategy for at least 1 database, including any limits used, such that it could be repeated.	Appendix 2
Selection of sources of evidence†	9	State the process for selecting sources of evidence (i.e., screening and eligibility) included in the scoping review.	5
Data charting process‡	10	Describe the methods of charting data from the included sources of evidence (e.g., calibrated forms or forms that have been tested by the team before their use, and whether data charting was done independently or in duplicate) and any processes for obtaining and confirming data from investigators.	5-6
Data items	11	List and define all variables for which data were sought and any assumptions and simplifications made.	5
Critical appraisal of individual sources of evidence§	12	If done, provide a rationale for conducting a critical appraisal of included sources of evidence; describe the methods used and how this information was used in any data synthesis (if appropriate).	n/a
Synthesis of results	13	Describe the methods of handling and summarizing the data that were charted.	5-6
**RESULTS**			

For peer review only - http://bmjopen.bmj.com/site/about/guidelines.xhtml

SECTION	ITEM	PRISMA-ScR CHECKLIST ITEM	REPORTED ON PAGE #
Selection of sources of evidence	14	Give numbers of sources of evidence screened, assessed for eligibility, and included in the review, with reasons for exclusions at each stage, ideally using a flow diagram.	Appendix 2
Characteristics of sources of evidence	15	For each source of evidence, present characteristics for which data were charted and provide the citations.	6, References
Critical appraisal within sources of evidence	16	If done, present data on critical appraisal of included sources of evidence (see item 12).	n/a
Results of individual sources of evidence	17	For each included source of evidence, present the relevant data that were charted that relate to the review questions and objectives.	6
Synthesis of results	18	Summarize and/or present the charting results as they relate to the review questions and objectives.	7-8
**DISCUSSION**			
Summary of evidence	19	Summarize the main results (including an overview of concepts, themes, and types of evidence available), link to the review questions and objectives, and consider the relevance to key groups.	8
Limitations	20	Discuss the limitations of the scoping review process.	9
Conclusions	21	Provide a general interpretation of the results with respect to the review questions and objectives, as well as potential implications and/or next steps.	10
**FUNDING**			
Funding	22	Describe sources of funding for the included sources of evidence, as well as sources of funding for the scoping review. Describe the role of the funders of the scoping review.	10

PRISMA-ScR = Preferred Reporting Items for Systematic reviews and Meta-Analyses extension for Scoping Reviews.
* Where *sources of evidence* (see second footnote) are compiled from, such as bibliographic databases, social media platforms, and Web sites.
† A more inclusive/heterogeneous term used to account for the different types of evidence or data sources (e.g., quantitative and/or qualitative research, expert opinion, and policy documents) that may be eligible in a scoping review as opposed to only studies. This is not to be confused with *information sources* (see first footnote).
‡ The frameworks by Arksey and O'Malley (6) and Levac and colleagues (7) and the JBI guidance (4, 5) refer to the process of data extraction in a scoping review as data charting.
§ The process of systematically examining research evidence to assess its validity, results, and relevance before using it to inform a decision. This term is used for items 12 and 19 instead of "risk of bias" (which is more applicable to systematic reviews of interventions) to include and acknowledge the various sources of evidence that may be used in a scoping review (e.g., quantitative and/or qualitative research, expert opinion, and policy document).

From: Tricco AC, Lillie E, Zarin W, O'Brien KK, Colquhoun H, Levac D, et al. PRISMA Extension for Scoping Reviews (PRISMAScR): Checklist and Explanation. Ann Intern Med. 2018;169:467–473. doi: 10.7326/M18-0850.

For peer review only - http://bmjopen.bmj.com/site/about/guidelines.xhtml

Appendix 2: Inclusion/Exclusion and Search Strategy

	Inclusion	Exclusion
**Medical Abortion**	• Medical Abortion<br>• Misoprostol, mifepristone, combipack and/or oxytocin	• Abortion services<br>• Contraception<br>• Reproductive health or family planning supplies without mentioning abortion medications
**Covid-19**	• All terms related to the SARS-COV-2 pandemic	• Previous SARS outbreaks<br>• Other pandemics or epidemics
**Procurement**	• Characteristics of ordering and/or receiving medical abortion medicines<br>• Examples of adapting to medical abortion medicine supply disruption<br>• Characteristics or examples of medical abortion supply chain disruption affecting procurement	• Procurement of commodities not related to medical abortion
**Evidence base**	• Primary data provided<br>• Methodological basis for study	• Opinion or commentary<br>• Speculative content

Search strategy

**Type of studies**	All; no protocols
**Condition**	Unwanted pregnancy
**Time frame**	January 1, 2020 to April 12, 2022
**Type of publications**	Published and unpublished articles
**Language**	English
**Setting**	All settings

For peer review only

WHO COVID Database

Retrieved April 14, 2022

#	Searches
1. Medical Abortion	tw:"medical abortion" OR tw:"medication abortion" OR mh:misoprostol or mh:mifepristone OR tw:misoprostol OR tw:mifepristone OR mh:"postcoital contraception" OR tw:"postcoital contraception" OR tw:"emergency contraception" OR tw:"abortifacient agents" OR tw:mifegyne OR tw:mifégyne OR tw:mifeprex OR tw:cytotec OR tw:glefos OR tw:amethopterin OR tw:cityl OR tw:cyprostol OR tw:cytolog OR tw:gastotec OR tw:gastrul OR tw:gymiso OR tw:hemoprostol OR tw:isprelor OR tw:misel OR tw:misodel OR tw:misopress OR tw:misoprostil)
2. Covid-19	tw:"corona virus" OR " tw:corona pandemic" OR tw:coronavir* OR tw:"sars virus" OR tw:COVID OR tw:Covid-19 OR tw:pandemic
#1 AND #2 New Articles from WHO Database	380

Pubmed

Retrieved April 14, 2022

#	Searches
1. Medical Abortion	"Contraceptives, Postcoital"[MeSH] OR "medical abortion"[tw] OR "medication abortion"[tw] OR "Abortifacient Agents" [MeSH] OR Misoprostol[MeSH] OR "Mifepristone"[MeSH] OR Mifegyne[TW] OR Mifégyne[TW] OR Mifeprex[TW] OR Mifepristone[TW] OR amethopterin[TW] OR cityl[TW] OR cyprostol[TW] OR cytolog[TW] OR gastotec[TW] OR gastrul[TW] OR gymiso[TW] OR hemoprostol[TW] OR isprel[TW] OR misel[TW] OR misodel[TW] OR misopress[TW] OR misoprostil[TW] OR misotrol[TW] OR misoclear[TW] OR mysodelle[tw] OR “R 38486”[tw] OR R38486[tw] OR “RU 38486”[tw] OR “RU 486”[tw] OR RU38486 [tw] OR RU486[tw] OR RU-486[tw] OR ZK 98296 [tw] OR ZK98296 [TW] OR "misoprostol"[MeSH Terms] OR misoprostol[tw] OR Cytotec[TW] OR Glefos[TW] OR “SC 29333”[TW] OR “SC 30249”[TW] OR SC29333[TW] OR SC30249 [TW] OR Amethopterin [ALL] OR Mexateè[ALL] OR (("Prostaglandins E, Synthetic"[MeSH] OR "methotrexate"[MeSH]) AND ("abortion, induced"[MeSH] OR “Abortion, Criminal"[MeSH] OR "Abortion, Septic"[MeSH] OR "Abortion Applicants"[MeSH] OR "abortion, missed"[MeSH] OR "abortion, legal"[MeSH])) OR (”family planning”[tw] AND (suppl*[TW] OR commodity[TW] OR drugs[TW] OR products[TW]))
2.	"corona virus"[tiab] OR "corona pandemic"[tiab] OR coronavir*[tiab] OR betacoronavir*[tiab] OR covid19[tiab] OR covid*[tiab] OR "wuhan

For peer review only

Covid-19	virus"[tiab] OR "severe acute respiratory syndrome"[tiab] OR "novel CoV"[tiab] OR nCoV*[tiab] OR hcov[tiab] OR "CoV 2"[tiab] OR CoV2[tiab] OR 2019nCoV[tiab] OR NCOV19[tiab] OR ncovid[tiab] OR sarscov2[tiab] OR sars2[tiab] OR "sars 2"[tiab] OR "sars-cov"[tiab] OR "sarscov 2"[tiab] OR "sars cov 2"[tiab] OR "sars co v 2"[tiab] OR sarscov[tiab] OR "sars virus"[tiab] OR COVID-19[MeSH]
#1 AND #2 New Articles from PubMed	278

Science Direct

Retrieved April 14, 2022

#	Searches
1. Medical Abortion (limit of 8 boolean operations/search)	1)"medical abortion"[tw] OR "medication abortion"[tw] OR misoprostol[tw] OR mifepristone[tw] OR "postcoital contraception"[tw] OR "emergency contraception"[tw] OR "abortifacient agents"[tw]<br><br>2)mifegyne[TW] OR mifégyne[TW] OR mifeprex[TW] OR cytotec [TW] OR glefos[TW] OR amethopterin[TW] OR cityl[TW] OR cyprostol[tw]<br><br>3)cytolog OR gastotec OR gastrul OR gymiso OR hemoprostol OR isprelor OR misel OR misodel<br><br>4) misopress OR misoprostil OR misotrol OR misoclear OR mysodelle OR R38486 OR ”RU 38486”<br><br>5) ”RU 486” OR RU38486 OR RU486 OR RU-486 OR ”ZK 98296” OR ZK98296 OR (”family planning” AND (suppl* OR commodity OR drugs OR products))
2. Covid-19	Covid-19
#1 AND #2 New Articles from Science Direct	135

PMC (Pubmed Central)

Retrieved April 14, 2022

#	Searches
1. Medical Abortion	"Contraceptives, Postcoital"[MeSH] OR "medical abortion"[tw] OR "medication abortion"[tw] OR "Abortifacient Agents" [MeSH] OR Misoprostol[MeSH] OR misoprostol[tw] OR "Mifepristone"[MeSH] OR

	Mifegyne[tw] OR Mifégyne[tw] OR Mifeprex[tw] OR Mifepristone[tw] OR amethopterin[tw] OR cityl[tw] OR cyprostol[tw] OR cytolog[tw] OR gastotec[tw] OR gastrul[tw] OR gymiso[tw] OR hemoprostol[tw] OR isprel[tw] OR misel[tw] OR misodel[tw] OR misopress[tw] OR misoprostil[tw] OR misotrol[tw] OR misoclear[tw] OR mysodelle[tw]OR Cytotec[tw] OR Glefos[tw])
2. Covid-19	"corona virus"[tw] OR "corona pandemic"[tw] OR coronavir*[tw] OR betacoronavir*[tw] OR covid19[tw] OR covid*[tw] OR "wuhan virus"[tw] OR "severe acute respiratory syndrome"[tw] OR "novel CoV"[tw] OR nCoV*[tw] OR hcov[tw] OR "CoV 2"[tw] OR CoV2[tw] OR 2019nCoV[tw] OR NCOV19[tw] OR ncovid[tw] OR sarscov2[tw] OR sars2[tw] OR "sars 2"[tw] OR "sars-cov"[tw] OR "sarscov 2"[tw] OR "sars cov 2"[tw] OR "sars co v 2"[tw] OR sarscov[tw] OR "sars virus"[tw] OR COVID-19[MeSH]
#1 AND #2 New Articles from PMC	501

Cochrane Library

Retrieved April 14, 2022

#	Searches
1. Medical Abortion	#1MeSH descriptor: [Abortion, Induced] explode all trees<br><br>#2("medical abortion" OR "medication abortion" or mifepristone OR misoprostol OR ”postcoital contraception” OR ”emergency contraception” OR "abortifacient agents" OR mifegyne OR mifégyne OR mifeprex OR cytotec OR glefos):ti,ab,kw (Word variations have been searched)<br><br>#3”family planning” AND (suppl* OR commodity OR drugs OR products)
2. Covid-19	#4coronavirus 2019 OR covid OR coronavir* OR betacoronavir* OR covid19 OR covid* OR “wuhan virus” OR “severe acute respiratory syndrome” OR “novel CoV” OR nCoV* OR hcov OR CoV 2 OR CoV2 OR 2019nCoV OR NCOV19 OR sarscov2 OR sars2 OR sars 2 OR sars-cov OR “sarscov 2” OR “sars cov 2” OR “sars co v 2” OR sarscov OR “sars virus” OR COVID OR Covid-19 OR pandemic<br><br>#5MeSH descriptor: [SARS-CoV-2] explode all trees
(#1 OR #2 OR #3) AND (#4 AND #5) New Articles from the Cochrane Library:	8 [Removed 7 protocols)

For peer review only

EMBASE

Retrieved April 14, 2022

#	Searches
1. Medical Abortion	('contraceptives, postcoital':ti,ab,kw OR 'medical abortion':ti,ab,kw OR 'medication abortion':ti,ab,kw OR 'abortifacient agents':ti,ab,kw OR 'mifepristone':ti,ab,kw OR mifegyne:ti,ab,kw OR mifégyne:ti,ab,kw OR mifeprex:ti,ab,kw OR mifepristone:ti,ab,kw OR amethopterin:ti,ab,kw OR cityl:ti,ab,kw OR cyprostol:ti,ab,kw OR cytolog:ti,ab,kw OR gastotec:ti,ab,kw OR gastrul:ti,ab,kw OR gymiso:ti,ab,kw OR hemoprostol:ti,ab,kw OR isprel:ti,ab,kw OR misel:ti,ab,kw OR misodel:ti,ab,kw OR misopress:ti,ab,kw OR misoprostil:ti,ab,kw OR misotrol:ti,ab,kw OR misoclear:ti,ab,kw OR misoprostol:ti,ab,kw)
2. Covid-19	'coronavirus disease 2019'
#1 AND #2 New Articles from EMBASE	71

For peer review only

BMJ Open

Disruptions to the procurement of medical abortion medicines during COVID-19: a scoping review

Journal:	*BMJ Open*
Manuscript ID	bmjopen-2022-064848.R2
Article Type:	Original research
Date Submitted by the Author:	05-Oct-2022
Complete List of Authors:	Cassinath, Natasha ; World Health Organization, Sexual and Reproductive Health and Research and UNDP-UNFPA-UNICEF-WHO-World Bank Special Programme of Research, Development and Research Training in Human Reproduction (HRP),<br>TITULAER , Patricia ; World Health Organization, Sexual and Reproductive Health and Research and UNDP-UNFPA-UNICEF-WHO-World Bank Special Programme of Research, Development and Research Training in Human Reproduction (HRP),<br>Läser, Laurence; World Health Organization, Sexual and Reproductive Health and Research and UNDP-UNFPA-UNICEF-WHO-World Bank Special Programme of Research, Development and Research Training in Human Reproduction (HRP),<br>Lavelaneta, Antonella ; World Health Organization, Department of Sexual and Reproductive Health and Research and UNDP-UNFPA-UNICEF-WHO-World Bank Special Programme of Research, Development and Research Training in Human Reproduction (HRP),<br>Ahsan, Safia; Reproductive Health Supplies Coalition<br>Toedtli, Francelle ; United Nations Population Fund<br>Mawa , Stephen ; United Nations Population Fund<br>Rehnstrom Loi, Ulrika; World Health Organization, Sexual and Reproductive Health and Research and UNDP-UNFPA-UNICEF-WHO-World Bank Special Programme of Research, Development and Research Training in Human Reproduction (HRP),
**Primary Subject Heading**:	Reproductive medicine
Secondary Subject Heading:	Obstetrics and gynaecology, Qualitative research
Keywords:	COVID-19, PUBLIC HEALTH, Reproductive medicine < GYNAECOLOGY

SCHOLARONE™
Manuscripts

I, the Submitting Author has the right to grant and does grant on behalf of all authors of the Work (as defined in the below author licence), an exclusive licence and/or a non-exclusive licence for contributions from authors who are: i) UK Crown employees; ii) where BMJ has agreed a CC-BY licence shall apply, and/or iii) in accordance with the terms applicable for US Federal Government officers or employees acting as part of their official duties; on a worldwide, perpetual, irrevocable, royalty-free basis to BMJ Publishing Group Ltd ("BMJ") its licensees and where the relevant Journal is co-owned by BMJ to the co-owners of the Journal, to publish the Work in this journal and any other BMJ products and to exploit all rights, as set out in our licence.

The Submitting Author accepts and understands that any supply made under these terms is made by BMJ to the Submitting Author unless you are acting as an employee on behalf of your employer or a postgraduate student of an affiliated institution which is paying any applicable article publishing charge ("APC") for Open Access articles. Where the Submitting Author wishes to make the Work available on an Open Access basis (and intends to pay the relevant APC), the terms of reuse of such Open Access shall be governed by a Creative Commons licence – details of these licences and which Creative Commons licence will apply to this Work are set out in our licence referred to above.

Other than as permitted in any relevant BMJ Author's Self Archiving Policies, I confirm this Work has not been accepted for publication elsewhere, is not being considered for publication elsewhere and does not duplicate material already published. I confirm all authors consent to publication of this Work and authorise the granting of this licence.

For peer review only

Title

Disruptions to the procurement of medical abortion medicines during COVID-19: a scoping review

Authors

Natasha Cassinath[1], Patricia Titulaer[1], Laurence Läser[1], Antonella Lavelaneta[1], Safia Ahsan[2], Francelle Kwankam Toedtli[3], Stephen Mawa[4], Ulrika Rehnström Loi[1]*

1. UNDP-UNFPA-UNICEF-WHO-World Bank Special Programme of Research, Development and Research Training in Human Reproduction (HRP), Department of Sexual and Reproductive Health and Research, World Health Organization, Geneva, Switzerland

2. Reproductive Health Supplies Coalition, Washington DC, USA

3. UNFPA, New York, New York, USA

4. UNFPA, Supply Chain Management Unit, Copenhagen, Denmark

*Corresponding author: Ulrika Rehnström Loi: rehnstromu@who.int

Word Count: 3506 (without abstract, boxes, tables and figures, references, or appendices)

For peer review only

ABSTRACT

Objectives

This scoping review aimed to systematically search, retrieve, and map the extent and characteristics of available literature on the evidenced disruptions to medical abortion (MA) medicine procurement caused by the COVID-19 outbreak.

Design

Scoping review using Arksey and O'Malley's methodology and Levac et al's methodological enhancement with adherence to the PRISMA extension for scoping reviews.

Data Sources

PUBMED, EMBASE, PMC, Science Direct, the Cochrane Library, and Google Scholar were searched from January 2020 to April 2022.

Eligibility Criteria

We included articles in English that (1) contained information on MA medicines: (2) included descriptions of procurement disruptions, including those with examples, characteristics and/or statistics; (3) documented events during the COVID-19 pandemic; and 4) presented primary data.

Data Extraction and Synthesis

Two reviewers independently screened search results, performed a full text review of preliminarily included articles, and completed data extraction in a standard Excel spreadsheet. Extracted data from was compared for validation and synthesised qualitatively.

Results

The two articles included are unpublished grey literature demonstrating evidence of short-lived disruptions in sexual and reproductive health commodity procurement, including MA medicines, in Sub-Saharan Africa during the early months of the pandemic. Findings from two included grey literature articles show that in Sub-Saharan contexts, emergency preparedness, stockpiling, adaptations, and flexibility of key actors, including donors, alleviated Covid-19 disruptions allowing for resumption of services within weeks.

Conclusion

For peer review only

There is a need for increased empirical evidence of MA procurement challenges to understand which barriers to MA procurement may persist and impact continuity of supply while others can fuel resilience and preparedness efforts at the country and sub-regional levels. The lack of evidence from social marketing organizations and their networks is a significant gap as these actors constitute a vital artery in the distribution of MA commodities in low- and middle-income countries.

Keywords: procurement, medical abortion, COVID-19, supply chain, disruption, preparedness

Strengths and limitations of this study

- We considered 1,662 articles related to “medical abortion” and “covid-19”.
- The study uncovered critical areas for future medical abortion supply chain research.
- The available evidence for extraction was limited to two articles which were not published in peer reviewed journals.
- The articles reviewed address supply chain issues of Sub-Saharan African countries with only a brief discussion of procurement in each article and unclear distinction of challenges by type of procurer.
- As studies refer to sexual and reproductive health including medical abortion, there is a risk that some interventions or conclusions are erroneously attributed to the challenge of medical abortion procurement.

For peer review only

For peer review only - http://bmjopen.bmj.com/site/about/guidelines.xhtml

INTRODUCTION

Globally, roughly 73.3 million abortions take place per year, at least half of which are performed through medical abortion.[1,2] Medical abortion (MA) is a procedure that terminates pregnancy using medicines - either mifepristone and misoprostol or misoprostol alone.[3] Mifepristone is a powerful abortifacient that blocks progesterone receptors and glucocorticoid receptors in the placenta to stop growth. Misoprostol dilates the cervix and induces muscle contractions which clears the uterus. MA is widely considered to be a safe and effective means to terminate pregnancy.

Medical abortion medicines advanced the ability of women to obtain abortion safely, at home, and with limited or no interactions with healthcare providers, if desired. MA medicines figure on the World Health Organization (WHO) essential medicines list (EML) and are considered vital for comprehensive and post abortion care.[4,5] Country-based availability of MA medicines is restricted to where there is at least one legal ground for abortion. In 2019, 129 countries of a study of 158 had such a legal basis for abortion and, in principle, market authorization to stock and distribute MA medicines albeit in a controlled manner.[6]

Access to MA medicines is contingent on conducive policy, regulation, registration, EML inclusion, financing and supply chain management. When COVID-19 was declared a public health emergency of international concern by the WHO in January 2020, the restrictions in movement of goods and people strained supply chains and put the procurement of MA medicines in jeopardy. Procurement is the act of obtaining goods and services at an appropriate level of quality, at a specified time and location, and at a negotiated price.[7] It relies on established relationships with domestic and international suppliers; the capacity to accurately forecast demand; and the management of the supply pipeline to avoid stockouts, warehousing challenges or product expiry. All of these complex activities were reportedly compromised when domestic and international manufacturing, transport, donors, and regulations were impacted by COVID-19 containment efforts, just as MA procurement was needed more than ever.[8]

In some settings, MA medicines obtained by mail or locally with or without health provider assistance presented women with the option to bypass lockdown or distancing restrictions, and in others, accelerated the political acceptance of self-managed abortion as it became an urgent necessity for service continuity.[9,10,11] When the virus containment measures started to strain global supply chains and divert funds and energy to personal protective equipment (PPE), key stakeholders - armed with little information – anticipated that the procurement of MA

For peer review only - http://bmjopen.bmj.com/site/about/guidelines.xhtml

medicines was equally at risk and called for urgent action and preparedness, including stockpiling and close surveillance of the supply chains to offset potential procurement challenges.[12,13,14,15,16,17]

The UNDP-UNFPA-UNICEF-WHO-World Bank Special Programme of Research, Development and Research Training in Human Reproduction (HRP) conducted a pre-scoping survey to better understand the impact of COVID-19 on the ability to fund and procure MA medicines. There were twenty-two (22) respondents from United Nations Population Fund (UNFPA), non-governmental organisations (NGOs) and social marketing organizations . This resulted in a list of supply chain funding and procurement challenges, as well as successful adaptations. .However, no clear root causes of disruptions, pervasive challenges, or mitigation measures were identified. The rationale for this scoping review was to explore what emerging evidence on MA medicines procurement disruptions existed over the first two years of the pandemic and identify research gaps. Understanding the causes of MA supply chain fragility or resilience in the short and longer term is essential to the development of mechanisms to forecast and plan for reliable supply in uncertain future scenarios. This understanding is of interest to comprehensive abortion care stakeholders, including actors involved in MA supply procurement, risk management, emergency preparedness, and donors. Thus, the aim of this scoping review is to systematically search, retrieve and map the extent and characteristics of available literature on the disruptions to MA medicine procurement during the COVID-19 outbreak.

METHODS

Study design

We undertook a scoping review to explore the nature and extent of the evidence related to disruptions to procurement of MA medicines during COVID-19. We considered further areas of inquiry related to the types of disruption, causes and adaptations, but intentionally left the question broad to be as inclusive as possible. The methods for this review were informed by Arksey and O'Malley's methodology and Levac et al's methodological enhancement.[18,19]. This paper adheres to the Preferred Reporting Items for Systematic Reviews and Meta-Analyses (PRISMA) extension for scoping reviews.[20] The PRISMA checklist can be found in appendix 1.

Supplementary data

For peer review only

Appendix 1: PRISMA checklist

Patient and public involvement statement

During the planning phase we consulted partners and stakeholders through a virtual survey and several virtual meetings to engage them in the scoping review and support to make sense of the study findings.

Search strategy

We piloted different search strategies including the concepts of “medical abortion,” “procurement,” “covid-19” with no geographical limitations. One reviewer (NC) supported by four advisory team members (PT, LL, AL, UR) harvested keywords from similar studies and trialled these in PubMed. We developed the final search strategy in consultation with the WHO medical librarian. We reduced the number of concepts to two: “medical abortion” and “covid-19” and included studies from January 2020 (right before the pandemic was officially declared) to April 2022 (4th- 6th wave depending on location). The comprehensive search strategy is presented in appendix 2. We conducted a database search in PubMed, PubMed Central, Embase, Science Direct, the Cochrane Library and the WHO Covid-19 database between November 4-6, 2021 and refreshed it on April 12, 2022. We searched Google Scholar with additional filters (“supply” OR “supply chain” OR “disruption”) to manage the volume of results. Additional grey literature was obtained by searching target organizations’ websites (e.g., UNFPA, WHO, MSI Reproductive Choices, DKT International, Reproductive Health Supplies Coalition). The reviewers used forward and backward citation searching of all articles in the full text review stage to find any additional studies fitting the inclusion criteria. All search results were stored using EndNote™ reference manager software and uploaded to Covidence® systematic review software.[21,22]

Supplementary data

Appendix 2 – Search Strategy

Study selection and data extraction process

For peer review only - http://bmjopen.bmj.com/site/about/guidelines.xhtml

Two reviewers (NC, PT) screened all titles and abstracts for inclusion independently. Studies that appeared relevant in a first instance were subjected to a full text review. We included articles that met the following inclusion criteria: (1) contained information on MA medicines; (2) included descriptions of procurement disruptions, including those with examples, characteristics and/or statistics; (3) documented events during the COVID-19 pandemic; and 4) articles presenting primary data. These criteria were developed in consult with all team members, based on the preliminary results of the search. We considered all English language documents from the timespan referred to above. A full description of the search terms, results, and inclusion/exclusion criteria is found in Appendix 2.

We developed a data extraction form in a standard Excel spreadsheet. The data points included document and study characteristics: author(s), title, publication year, journal, country/region, aim, nature of disruptions, root causes, adaptations, and author's conclusions. Two reviewers independently conducted the data abstraction. Each reviewer's extraction was compiled into a single excel spreadsheet in Microsoft Excel for validation.[23]

Data synthesis

Categorized data in the extraction table (Table 1 and 2) was analyzed across studies to identify emerging themes and subthemes related to the research question. Debriefing sessions were held with two members of the team to improve rigour and reduce researcher bias. Limitations to the heterogeneity of results were acknowledged. Study quality was not assessed as this is not a requirement for scoping reviews.[18]

REVIEW FINDINGS

Figure 1 shows the PRISMA flow chart. The search strategy returned 1662 results from the search of 6 databases, google scholar and web search. Of these, we removed 816 duplicate records and excluded 811 records that did not meet the inclusion criteria. . We conducted a full text review of 35 articles. Ultimately, we extracted information from 2 documents.

For peer review only
For peer review only - http://bmjopen.bmj.com/site/about/guidelines.xhtml

Characteristics of Included Articles and Data Extraction

Table 1 outlines the characteristics of the two studies included in the scoping review. Both articles present national level data on supply chain disruptions to sexual and reproductive health (SRH) or family planning commodities including MA medicines. Table 2 outlines the data extracted from the selected articles.

For peer review only

Table 1: Study characteristics

Author(s) Year	Article type	Journal	Data Collection	Region	Aim	Commodity/ Medicine Discussed
RHSC 2021 [24]	Rapid Assessment	Grey literature	53 interviews conducted globally<br><br>Data collected March - October 2020*	Global, with additional quantitative data from Ethiopia, Zambia, and Kenya	To understand the disruptions to SRH supply chains during the pandemic and chart a roadmap for supply chain resiliency	General SRH including MA
Otieno et al 2021 [25]	Rapid Needs Assessment	Grey literature	Survey data from 17 African ministries of health/Data collected July 2020	17 Sub Saharan African Countries	To assess and document continuity of SRH services with a focus on safe abortion, post abortion care and family planning services during the COVID -19 pandemic	Family planning and MA

* This study included pre-pandemic data. Pre-pandemic and covid specific data were distinguishable.

Table 2: Data extraction - Disruption, Adaptation and Author's Conclusions

For peer review only - http://bmjopen.bmj.com/site/about/guidelines.xhtml

Author(s) Year	Types of Disruption	Adaptations	Authors' conclusions
RHSC 2021.[24]	Quarantine at ports/border closures; manufacturing stoppage; shortage of freight containers; increased product and freight costs; lack of air cargo/increased demand for sea freight; workforce reduction; reduced demand for MA; uncertainty due to changing restrictions; regulatory approvals and delay in reauthorization of products	Diversification of delivery channel; transition to sea freight; mapping and tracking adaptations; dispensing guidelines were changed; funder flexibility and responsiveness; Policy guidance from WHO and UN agencies helped overcome bottlenecks, particularly around the movement of health products and the inclusion of SRH within essential services.	Manufacturing, logistics and systems (including policies and procedures) were most affected. Data from six countries showed no unusual changes in stock levels. Higher initial stock levels mitigated delays. Within 2–4 months, most companies and organizations resumed operations with new constraints. Many health supply chains rely on material inputs from China and India, including active pharmaceutical ingredients. Transportation will continue to be challenging.
Otieno et al 2021.[25]	Limited public procurement; Funding shortfall; General stock delay	None related specifically to procurement	MA drugs available in health facilities in 13 (76%) countries. Where not available, reasons included: abortion forbidden, drugs not in health facilities but available in the pharmacies, controlled drugs not available for MA, available but for induction of labour and for post-partum haemorrhage, and abortion drugs not included on the essential medicines list. Madagascar had no abortion care due to lack of trained healthcare workers.

For peer review only

The findings from the included articles were organized into five areas related to system-level MA medicines procurement during the COVID-19 pandemic: 1) pre-pandemic stockout mitigation strategies and their effects during the pandemic; 2) procurement adaptations at the international and local levels made in response to the pandemic; 3) the impact of manufacturing and transport related disruptions to procurement; 4) enabling or disabling national policies put in place during the pandemic; and 5) cost increases and changes to budget management due to the pandemic.

For peer review only - http://bmjopen.bmj.com/site/about/guidelines.xhtml

Both articles described stockouts of SRH medicines as a regular occurrence in many countries pre-pandemic.[24,25] In some countries, this risk was mitigated by maintaining a large stockpile of some of these medicines. During COVID-19, this protective overstock may have served to lessen the levels of stockouts in the short term. However, by the end of 2020, there was evidence of increased stockout in Zambia, Ethiopia, and Kenya, where stocks had otherwise remained stable.[24] In other Sub-Saharan Africa countries, COVID-19 related stockouts of SRH commodities began in March 2020 or later and continued to occur until at least October 2020. During this period, procurers were faced with increased costs related to PPE and pandemic response, commodity purchase prices, and transport; limited time to negotiate or perform price comparisons; and the additional effort to secure the transport and fulfil the purchase orders. These hurdles were followed by the implementation of adaptations allowing for abortion services to return to pre-pandemic levels within “weeks, not months”.[24] Adaptations included attempting to identify alternative suppliers; adjusting to limited transport options; mapping and tracking orders; lobbying for funder flexibility and responsiveness; redistributing stock; and advocating SRH and/or MA supplies priority.[24] Diversifying the supplier base was a key challenge as manufacturers are largely geographically concentrated in Asia.[24]

At the onset of the pandemic, manufacturers stated that fluctuations in demand, anticipated supply chain disruptions, and panic buying challenged their ability to plan and produce SRH supplies in step with actual demand but they were able to rapidly return to pre-pandemic production levels once they adopted COVID-19 protocols in their work environment.[24]

Replenishment stock of SRH commodities through usual channels proved challenging, but in some instances there was evidence of local coordination and collaborative management of inventory between procurers, such as service providers and retailers, allowing for healthy stocks in one region to be redeployed to another.[25] WhatsApp was used to improve communication and support the coordination of stock availability and redistribution.[24,25]

National policies related to the movement of select goods and workers played a part in stalling the ability of procurers to receive MA medicines. When abortion was not immediately deemed an essential service, the flow of MA medicines was deprioritized and prone to delay.[25] When MA was designated as an essential medicine, it positively impacted the ability to receive and distribute MA medicines nationally.[24,25]

For peer review only - http://bmjopen.bmj.com/site/about/guidelines.xhtml

Increases in transport costs and the diversions of funds to PPE were found to have direct impacts on SRH and MA budgets.[24,25] However, the burden of these costs was not always carried by the procurer – manufacturers, wholesalers and donors also incurred a share of these costs.[24] In the short term, donor flexibility; budgetary reallocation from PPE to SRH commodities or vice versa; and/or the ability to increase prices downstream in the supply chain allowed for continued procurement. The WHO African Region found that health ministries' financing for national procurement was stable in 2020 as funding had been allocated and orders were already placed pre-pandemic.[25]

Concerns related to post-pandemic costs were raised in both articles.[24,25] Manufacturers claimed that improving risk mitigation efforts for the future, such as by maintaining larger stockpiles or developing relationships with a more diversified supplier base, would drive up their costs and affect product pricing.[24] Both articles highlighted that transport costs may continue to be impacted by backlogs, travel restrictions, storage constraints and limited freight options.[24,25]

DISCUSSION

While there exists no straightforward data regarding the prevalence of medical abortion administration, proxy information from studies looking at the readiness of 210 health facilities in 11 African countries to provide post abortion care demonstrates that the primary challenge is not the availability of uterotonics (misoprostol or oxytocin), but rather, limitations of staff and critical care unit infrastructure.[26]

Before the COVID-19 pandemic, unintended pregnancy rates were higher in low- and middle-income countries (respectively, 93 and 66 unintended pregnancies per 1000 reproductive-aged women per year) than in high-income countries (34 per 1000).[1] Before the pandemic, women in low and middle income countries were also less likely to have a safe and legal abortion. For example, the most recent data available indicates that in Sub Saharan Africa between 2015-2019, 8.0 million annual abortions occurred and between 2010-2014, 77% of abortions were unsafe.[2] Based on past experience of pandemics and other disruptive events, the magnitude of these disparities would be expected to increase the need and challenge the access to abortion services and products.[27]

Despite the anticipation of catastrophic impacts of COVID-19 on the supply of abortion medicines, this scoping review, employing a systematic comprehensive search strategy to

For peer review only - http://bmjopen.bmj.com/site/about/guidelines.xhtml

gather all types of available evidence, found an almost negligeable number of articles (2) on the topic, both grey literature.

The limited evidence uncovered that preparedness — namely buffer stocks— and rapid adaptation to new conditions by multiple stakeholders (donors, policy makers, manufacturers, NGOs) were key ingredients in ensuring the continuous supply of SRH commodities, including MA medicines.

Concerns around COVID-19 disruptions on family planning were justified based on past experience, but ultimately the available evidence indicates that the scale of disturbance in the select Sub Saharan countries was not as detrimental as was initially envisioned, and/or service returned to pre-pandemic levels once lockdown periods concluded. [28,29]

It remains to be known how expansively resilience was experienced, where, and how long it can be expected to hold given that continued COVID-19 risk mitigation measures, pre-emptive stockpiling, limited transport options, shipping container shortages and rising petrol prices will increase the cost of doing business. National health budgets will be stretched as COVID-19 testing and vaccination efforts continue, and funds may be redirected to general population health and mental health due to neglect over the last two years.[30,31] In addition, organizations or companies reliant on international donations and/or unable to absorb these increased costs are anticipated to face a tight financing landscape, particularly due to the reduced levels of funding for safe abortion worldwide.[32]

It cannot be overstated that MA commodity medicines are difficult to obtain in many parts of the world and that “returning to normal” does not imply a satisfactory status quo, particularly in restrictive jurisdictions or fragmented markets where data sets are unavailable.

Strengths and Limitations

The strengths associated with this scoping review are that it includes both published and un-published literature and provides an overview of current evidence in the field of MA medicine procurement caused by the COVID-19 outbreak. Our focus was strictly on MA procurement and therefore, the availability of MA medicines does not infer access by clients. However, there are several limitations to this review. The available evidence was limited and not published in peer reviewed journals. There is only a brief discussion of procurement in each article, in favour of a

For peer review only - http://bmjopen.bmj.com/site/about/guidelines.xhtml

broader discussion of supply chain issues affecting either SRH supplies broadly or family planning supplies specifically. The studies refer to SRH including MA, which made applicability of some interventions or conclusions difficult to attribute to MA procurement specifically. Importantly, the evidence from field-based nongovernmental organizations and global social marketing organizations is very limited in these studies. Social marketing organizations are recognized as important actors in the procurement and distribution of MA services and products in low- and middle-income countries yet no published documentation of their experiences with MA procurement during the COVID-19 pandemic was found. Additionally, the articles reviewed were heavily weighted towards the supply chain issues of Sub-Saharan African countries and may not be relevant or representative of the reality in geographies.

CONCLUSION

This scoping review identified evidence gaps and future research needs to 1) improve and increase the empirical evidence on MA medicines procurement and the greater supply chain, and 2) improve the resilience and preparedness of state and private health systems distributing MA medicines. Given the general nature of current research looking at multiple countries across multiple sexual and reproductive health products, further examination should be undertaken in countries, where market fragility is known to exist, to support risk mitigation and preparedness efforts. The most important contribution to this work would be in capturing the resilience of social marketing organizations and their network, who distribute the bulk of MA medicines in low- and middle-income countries worldwide. These organizations have outsized influence and experience in the procurement and distribution of MA products, particularly in low and middle income countries.[33]

Acknowledgements

The authors would like to acknowledge the input of Kavita Kothari, World Health Organization Librarian, Geneva, Switzerland for her suggestions and support in refining the search strategy.

For peer review only

For peer review only - http://bmjopen.bmj.com/site/about/guidelines.xhtml

Contributorship statement

The scoping review was conceptualized by URL, PT, SA and FKT. NC and PT conducted the review, including assessing literature and data synthesis. NC prepared the first draft manuscript with significant intellectual contributions from PT, AL, LL, SA, FKT, SM and URL. All authors that also reviewed and edited versions of the manuscript. The authors alone are responsible for the views expressed in this article, and they do not necessarily represent the views, decisions, or policies of the institutions with which they are affiliated. All authors approved the final manuscript for publication.

Competing interests

None declared.

Funding

This work was supported by the UNDP-UNFPA-UNICEF-WHO-World Bank Special Programme of Research, Development and Research Training in Human Reproduction (HRP), a cosponsored programme executed by the World Health Organization (WHO).

Data Sharing Statement

No additional data available.

For peer review only - http://bmjopen.bmj.com/site/about/guidelines.xhtml

Disclaimer

The views expressed in this article are those of the authors and do not necessarily represent the views of, and should not be attributed to, the World Health Organization, or the United Nations Population Fund.

For peer review only

Patient consent for publication

Not required

Ethics approval

An ethics statement is not applicable because this study was based exclusively on published literature.

Figure legend

Figure 1. PRISMA flow chart

For peer review only - http://bmjopen.bmj.com/site/about/guidelines.xhtml

References

1 Bearak J, Popinchalk A, Ganatra B, et al. Unintended pregnancy and abortion by income, region, and the legal status of abortion: estimates from a comprehensive model for 1990–2019. Lancet Glob Health 2020. doi: 10.1016/S2214-109X(20)30315-6.

2 Bankole A, Remez L, Owolabiet O, al., From Unsafe to Safe Abortion in Sub-Saharan Africa: Slow but Steady Progress, New York: Guttmacher Institute, 2020, https://www.guttmacher.org/report/ from-unsafe-to-safe-abortion-in-subsaharan-africa. doi:10.1363/2020.32446

3 Aronson JK, editor. Meyler's Side Effects of Drugs, Sixteenth Editions. 2016.

4 WHO. World Health Organization Model List of Essential Medicines – 22nd List, 2021. World Health Organization[Internet]. 2021.

5 WHO. Abortion care guideline. World Health Organization[Internet]. 2022.

6 Lavelanet, A.F., Schlitt, S., Johnson *et al.* Global Abortion Policies Database: a descriptive analysis of the legal categories of lawful abortion. *BMC Int Health Hum Rights* 18, 44 2018. doi.org/10.1186/s12914-018-0183-1

7 Turner RW. Supply management and procurement: From the basics to best-in-class. *J. Ross Publishing*; 2011 Oct 10.

8 IPPF. The role of quality medical abortion during the coronavirus crisis. IPPF[Internet]. June 2020

9 Skuster P, Dhillon J and Li J. Easing of Regulatory Barriers to Telemedicine Abortion in Response to COVID-19. *Front Glob Womens Health.* 2021. doi: 10.3389/FGWH.2021.705611

For peer review only

10 Todd-Gher J and Shah P. Abortion in the context of COVID-19: a human rights imperative, *Sex Reprod Health Matters*. 2020; 28:1, doi: 10.1080/26410397.2020.1758394

11 Kumar M, Daly M, De Plecker E, et al. Now is the time: a call for increased access to contraception and safe abortion care during the COVID-19 pandemic *BMJ Global Health* 2020;5:e003175.

12 Purdy C. How will COVID-19 affect global access to contraceptives–and what can we do about it? DevEx [Internet]. 2020 Mar 11.

13 Casas X. Protecting Women's Reproductive Health During the Pandemic. Human Rights Watch [Internet]. 2020 May 21.

14 Weinberger M, Hayes B, White J, et al. Doing Things Differently: What It Would Take to Ensure Continued Access to Contraception During COVID-19. *Glob Health Sci Pract.* 2020;8(2):169-75.

15 UNFPA. New UNFPA projections predict calamitous impact on women's health as COVID-19 pandemic continues. UNFPA [Internet]. April 2020

16 Women's Refugee Council. Disruptions and Adaptations: The Effects of COVID-19 on Contraceptive Services across the Humanitarian-Development Nexus. Women's Refugee Council [Internet]. December 2020.

17 Samandari G, Kapp N, Hamon C, et al. Challenges in the abortion supply chain: a call to action for evaluation research. *Reprod Health*. 2021;18.

18 Arksey H, O'Malley L. Scoping studies: towards a methodological framework. *Int J Soc Res* Methodol. 2005;8(1):19–32.

For peer review only - http://bmjopen.bmj.com/site/about/guidelines.xhtml

19 Levac D, Colquhoun H, O'Brien KK. Scoping studies: advancing the methodology. *Implement Sci.* 2010;5(1):1-9.

20 Tricco AC, Lillie E, Zarin W, et al. PRISMA Extension for Scoping Reviews (PRISMA-ScR): checklist and explanation. *Ann Intern Med* 2018 Oct 2;169(7):467-473.

21 The EndNote Team. Endnote 20. Clarivate. 2013.

22 Covidence systematic review software, Veritas Health Innovation, Melbourne, Australia. Available at www.covidence.org

23 Microsoft Corporation. Microsoft Excel [Internet]. 2018. Available from: https://office.microsoft.com/excel

24 Reproductive Health Supplies Coalition. Building Resilient Sexual and Reproductive Health Supply Chains During Covid-19 and Beyond. RHSC [Internet]. April 2021.

25 Otieno GO, Ouedraogo L, Nkurunziza T, et al. Continuity of Essential Sexual and Reproductive Health Services During Covid-19 Pandemic in the Who African Region. WHO [Internet]. 2021.

26 Compaoré, R, Mehrtash, H, Calvert, C, et al. Health facilities' capability to provide comprehensive postabortion care in Sub-Saharan Africa: Evidence from a cross-sectional survey across 210 high-volume facilities. Int J Gynecol Obstet. 2022; 156(Suppl. 1): 7– 19. doi:10.1002/ijgo.14056

27 Wenham C, Smith J, Davies SE, et al. Women are most affected by pandemics- lessons from past outbreaks. Nature. 2020. doi: 10.1038/d41586-020-02006-z

For peer review only

For peer review only - http://bmjopen.bmj.com/site/about/guidelines.xhtml

28 Banke-Thomas, A., Yaya, S. Looking ahead in the COVID-19 pandemic: emerging lessons learned for sexual and reproductive health services in low- and middle-income countries. Reprod Health.2021. doi:10.1186/s12978-021-01307-4

29 Wood SN, Karp C, OlaOlorun F, et al. Need for and use of contraception by women before and during COVID-19 in four sub-Saharan African geographies: results from population-based national or regional cohort surveys. *The Lancet Global Health.* 2021 Jun 1;9(6):e793-801.

30 Findling MG, Blendon RJ, Benson JM. Delayed Care with Harmful Health Consequences—Reported Experiences from National Surveys During Coronavirus Disease 2019. *JAMA.* 2020;1(12):e201463. doi:10.1001/jamahealthforum.2020.1463

31 Cutler DM, Summers LH. The COVID-19 Pandemic and the $16 Trillion Virus. *JAMA.* 2020;324(15):1495–1496. doi:10.1001/jama.2020.19759

32 McVeigh K. UK accused of 'abandoning' women with cuts to aid. The Guardian [Internet]. April 2021.

33 Mann Global Health. Landscape assessment: leveraging the role of national distributors to increase access to MA combi-packs in Africa [Internet]. 2019.

For peer review only - http://bmjopen.bmj.com/site/about/guidelines.xhtml

Figure 1. PRISMA flow chart

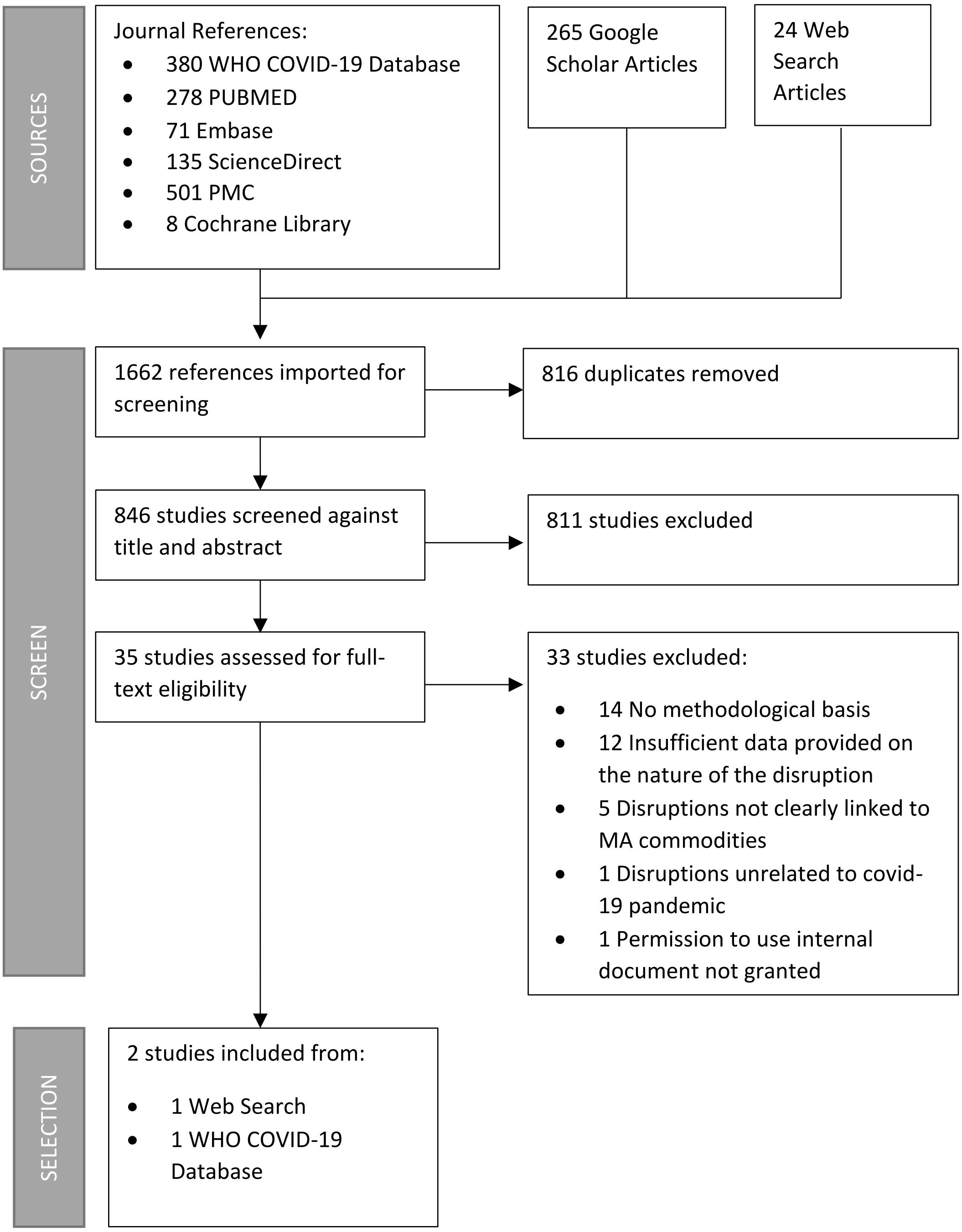

Appendix 1: Preferred Reporting Items for Systematic reviews and Meta-Analyses extension for Scoping Reviews (PRISMA-ScR) Checklist

SECTION	ITEM	PRISMA-ScR CHECKLIST ITEM	REPORTED ON PAGE #
**TITLE**			
Title	1	Identify the report as a scoping review.	1
**ABSTRACT**			
Structured summary	2	Provide a structured summary that includes (as applicable): background, objectives, eligibility criteria, sources of evidence, charting methods, results, and conclusions that relate to the review questions and objectives.	2
**INTRODUCTION**			
Rationale	3	Describe the rationale for the review in the context of what is already known. Explain why the review questions/objectives lend themselves to a scoping review approach.	4
Objectives	4	Provide an explicit statement of the questions and objectives being addressed with reference to their key elements (e.g., population or participants, concepts, and context) or other relevant key elements used to conceptualize the review questions and/or objectives.	3
**METHODS**			
Protocol and registration	5	Indicate whether a review protocol exists; state if and where it can be accessed (e.g., a Web address); and if available, provide registration information, including the registration number.	4
Eligibility criteria	6	Specify characteristics of the sources of evidence used as eligibility criteria (e.g., years considered, language, and publication status), and provide a rationale.	4
Information sources*	7	Describe all information sources in the search (e.g., databases with dates of coverage and contact with authors to identify additional sources), as well as the date the most recent search was executed.	4
Search	8	Present the full electronic search strategy for at least 1 database, including any limits used, such that it could be repeated.	Appendix 2
Selection of sources of evidence†	9	State the process for selecting sources of evidence (i.e., screening and eligibility) included in the scoping review.	5
Data charting process‡	10	Describe the methods of charting data from the included sources of evidence (e.g., calibrated forms or forms that have been tested by the team before their use, and whether data charting was done independently or in duplicate) and any processes for obtaining and confirming data from investigators.	5-6
Data items	11	List and define all variables for which data were sought and any assumptions and simplifications made.	5
Critical appraisal of individual sources of evidence§	12	If done, provide a rationale for conducting a critical appraisal of included sources of evidence; describe the methods used and how this information was used in any data synthesis (if appropriate).	n/a
Synthesis of results	13	Describe the methods of handling and summarizing the data that were charted.	5-6
**RESULTS**			

For peer review only

SECTION	ITEM	PRISMA-ScR CHECKLIST ITEM	REPORTED ON PAGE #
Selection of sources of evidence	14	Give numbers of sources of evidence screened, assessed for eligibility, and included in the review, with reasons for exclusions at each stage, ideally using a flow diagram.	Appendix 2
Characteristics of sources of evidence	15	For each source of evidence, present characteristics for which data were charted and provide the citations.	6, References
Critical appraisal within sources of evidence	16	If done, present data on critical appraisal of included sources of evidence (see item 12).	n/a
Results of individual sources of evidence	17	For each included source of evidence, present the relevant data that were charted that relate to the review questions and objectives.	6
Synthesis of results	18	Summarize and/or present the charting results as they relate to the review questions and objectives.	7-8
**DISCUSSION**			
Summary of evidence	19	Summarize the main results (including an overview of concepts, themes, and types of evidence available), link to the review questions and objectives, and consider the relevance to key groups.	8
Limitations	20	Discuss the limitations of the scoping review process.	9
Conclusions	21	Provide a general interpretation of the results with respect to the review questions and objectives, as well as potential implications and/or next steps.	10
**FUNDING**			
Funding	22	Describe sources of funding for the included sources of evidence, as well as sources of funding for the scoping review. Describe the role of the funders of the scoping review.	10

PRISMA-ScR = Preferred Reporting Items for Systematic reviews and Meta-Analyses extension for Scoping Reviews.
* Where *sources of evidence* (see second footnote) are compiled from, such as bibliographic databases, social media platforms, and Web sites.
† A more inclusive/heterogeneous term used to account for the different types of evidence or data sources (e.g., quantitative and/or qualitative research, expert opinion, and policy documents) that may be eligible in a scoping review as opposed to only studies. This is not to be confused with *information sources* (see first footnote).
‡ The frameworks by Arksey and O'Malley (6) and Levac and colleagues (7) and the JBI guidance (4, 5) refer to the process of data extraction in a scoping review as data charting.
§ The process of systematically examining research evidence to assess its validity, results, and relevance before using it to inform a decision. This term is used for items 12 and 19 instead of "risk of bias" (which is more applicable to systematic reviews of interventions) to include and acknowledge the various sources of evidence that may be used in a scoping review (e.g., quantitative and/or qualitative research, expert opinion, and policy document).

From: Tricco AC, Lillie E, Zarin W, O'Brien KK, Colquhoun H, Levac D, et al. PRISMA Extension for Scoping Reviews (PRISMAScR): Checklist and Explanation. Ann Intern Med. 2018;169:467–473. doi: 10.7326/M18-0850.

Appendix 2: Inclusion/Exclusion and Search Strategy

	Inclusion	Exclusion
**Medical Abortion**	• Medical Abortion<br>• Misoprostol, mifepristone, combipack and/or oxytocin	• Abortion services<br>• Contraception<br>• Reproductive health or family planning supplies without mentioning abortion medications
**Covid-19**	• All terms related to the SARS-COV-2 pandemic	• Previous SARS outbreaks<br>• Other pandemics or epidemics
**Procurement**	• Characteristics of ordering and/or receiving medical abortion medicines<br>• Examples of adapting to medical abortion medicine supply disruption<br>• Characteristics or examples of medical abortion supply chain disruption affecting procurement	• Procurement of commodities not related to medical abortion
**Evidence base**	• Primary data provided<br>• Methodological basis for study	• Opinion or commentary<br>• Speculative content

Search strategy

**Type of studies**	All; no protocols
**Condition**	Unwanted pregnancy
**Time frame**	January 1, 2020 to April 12, 2022
**Type of publications**	Published and unpublished articles
**Language**	English
**Setting**	All settings

For peer review only

WHO COVID Database

Retrieved April 14, 2022

#	Searches
1. Medical Abortion	tw:"medical abortion" OR tw:"medication abortion" OR mh:misoprostol or mh:mifepristone OR tw:misoprostol OR tw:mifepristone OR mh:"postcoital contraception" OR tw:"postcoital contraception" OR tw:"emergency contraception" OR tw:"abortifacient agents" OR tw:mifegyne OR tw:mifégyne OR tw:mifeprex OR tw:cytotec OR tw:glefos OR tw:amethopterin OR tw:cityl OR tw:cyprostol OR tw:cytolog OR tw:gastotec OR tw:gastrul OR tw:gymiso OR tw:hemoprostol OR tw:isprelor OR tw:misel OR tw:misodel OR tw:misopress OR tw:misoprostil)
2. Covid-19	tw:"corona virus" OR " tw:corona pandemic" OR tw:coronavir* OR tw:"sars virus" OR tw:COVID OR tw:Covid-19 OR tw:pandemic
#1 AND #2 New Articles from WHO Database	380

Pubmed

Retrieved April 14, 2022

#	Searches
1. Medical Abortion	"Contraceptives, Postcoital"[MeSH] OR "medical abortion"[tw] OR "medication abortion"[tw] OR "Abortifacient Agents" [MeSH] OR Misoprostol[MeSH] OR "Mifepristone"[MeSH] OR Mifegyne[TW] OR Mifégyne[TW] OR Mifeprex[TW] OR Mifepristone[TW] OR amethopterin[TW] OR cityl[TW] OR cyprostol[TW] OR cytolog[TW] OR gastotec[TW] OR gastrul[TW] OR gymiso[TW] OR hemoprostol[TW] OR isprel[TW] OR misel[TW] OR misodel[TW] OR misopress[TW] OR misoprostil[TW] OR misotrol[TW] OR misoclear[TW] OR mysodelle[tw] OR “R 38486”[tw] OR R38486[tw] OR “RU 38486”[tw] OR “RU 486”[tw] OR RU38486 [tw] OR RU486[tw] OR RU-486[tw] OR ZK 98296 [tw] OR ZK98296 [TW] OR "misoprostol"[MeSH Terms] OR misoprostol[tw] OR Cytotec[TW] OR Glefos[TW] OR “SC 29333”[TW] OR “SC 30249”[TW] OR SC29333[TW] OR SC30249 [TW] OR Amethopterin [ALL] OR Mexateè[ALL] OR (("Prostaglandins E, Synthetic"[MeSH] OR "methotrexate"[MeSH]) AND ("abortion, induced"[MeSH] OR “Abortion, Criminal"[MeSH] OR "Abortion, Septic"[MeSH] OR "Abortion Applicants"[MeSH] OR "abortion, missed"[MeSH] OR "abortion, legal"[MeSH])) OR (”family planning”[tw] AND (suppl*[TW] OR commodity[TW] OR drugs[TW] OR products[TW]))
2.	"corona virus"[tiab] OR "corona pandemic"[tiab] OR coronavir*[tiab] OR betacoronavir*[tiab] OR covid19[tiab] OR covid*[tiab] OR "wuhan

For peer review only

Covid-19	virus"[tiab] OR "severe acute respiratory syndrome"[tiab] OR "novel CoV"[tiab] OR nCoV*[tiab] OR hcov[tiab] OR "CoV 2"[tiab] OR CoV2[tiab] OR 2019nCoV[tiab] OR NCOV19[tiab] OR ncovid[tiab] OR sarscov2[tiab] OR sars2[tiab] OR "sars 2"[tiab] OR "sars-cov"[tiab] OR "sarscov 2"[tiab] OR "sars cov 2"[tiab] OR "sars co v 2"[tiab] OR sarscov[tiab] OR "sars virus"[tiab] OR COVID-19[MeSH]
#1 AND #2 New Articles from PubMed	278

Science Direct

Retrieved April 14, 2022

#	Searches
1. Medical Abortion (limit of 8 boolean operations/search)	1)"medical abortion"[tw] OR "medication abortion"[tw] OR misoprostol[tw] OR mifepristone[tw] OR "postcoital contraception"[tw] OR "emergency contraception"[tw] OR "abortifacient agents"[tw]<br><br>2)mifegyne[TW] OR mifégyne[TW] OR mifeprex[TW] OR cytotec [TW] OR glefos[TW] OR amethopterin[TW] OR cityl[TW] OR cyprostol[tw]<br><br>3)cytolog OR gastotec OR gastrul OR gymiso OR hemoprostol OR isprelor OR misel OR misodel<br><br>4) misopress OR misoprostil OR misotrol OR misoclear OR mysodelle OR R38486 OR ”RU 38486”<br><br>5) ”RU 486” OR RU38486 OR RU486 OR RU-486 OR ”ZK 98296” OR ZK98296 OR (”family planning” AND (suppl* OR commodity OR drugs OR products))
2. Covid-19	Covid-19
#1 AND #2 New Articles from Science Direct	135

PMC (Pubmed Central)

Retrieved April 14, 2022

#	Searches
1. Medical Abortion	"Contraceptives, Postcoital"[MeSH] OR "medical abortion"[tw] OR "medication abortion"[tw] OR "Abortifacient Agents" [MeSH] OR Misoprostol[MeSH] OR misoprostol[tw] OR "Mifepristone"[MeSH] OR

For peer review only

	Mifegyne[tw] OR Mifégyne[tw] OR Mifeprex[tw] OR Mifepristone[tw] OR amethopterin[tw] OR cityl[tw] OR cyprostol[tw] OR cytolog[tw] OR gastotec[tw] OR gastrul[tw] OR gymiso[tw] OR hemoprostol[tw] OR isprel[tw] OR misel[tw] OR misodel[tw] OR misopress[tw] OR misoprostil[tw] OR misotrol[tw] OR misoclear[tw] OR mysodelle[tw]OR Cytotec[tw] OR Glefos[tw])
2. Covid-19	"corona virus"[tw] OR "corona pandemic"[tw] OR coronavir*[tw] OR betacoronavir*[tw] OR covid19[tw] OR covid*[tw] OR "wuhan virus"[tw] OR "severe acute respiratory syndrome"[tw] OR "novel CoV"[tw] OR nCoV*[tw] OR hcov[tw] OR "CoV 2"[tw] OR CoV2[tw] OR 2019nCoV[tw] OR NCOV19[tw] OR ncovid[tw] OR sarscov2[tw] OR sars2[tw] OR "sars 2"[tw] OR "sars-cov"[tw] OR "sarscov 2"[tw] OR "sars cov 2"[tw] OR "sars co v 2"[tw] OR sarscov[tw] OR "sars virus"[tw] OR COVID-19[MeSH]
#1 AND #2 New Articles from PMC	501

Cochrane Library

Retrieved April 14, 2022

#	Searches
1. Medical Abortion	#1MeSH descriptor: [Abortion, Induced] explode all trees<br><br>#2("medical abortion" OR "medication abortion" or mifepristone OR misoprostol OR ”postcoital contraception” OR ”emergency contraception” OR "abortifacient agents" OR mifegyne OR mifégyne OR mifeprex OR cytotec OR glefos):ti,ab,kw (Word variations have been searched)<br><br>#3”family planning” AND (suppl* OR commodity OR drugs OR products)
2. Covid-19	#4coronavirus 2019 OR covid OR coronavir* OR betacoronavir* OR covid19 OR covid* OR “wuhan virus” OR “severe acute respiratory syndrome” OR “novel CoV” OR nCoV* OR hcov OR CoV 2 OR CoV2 OR 2019nCoV OR NCOV19 OR sarscov2 OR sars2 OR sars 2 OR sars-cov OR “sarscov 2” OR “sars cov 2” OR “sars co v 2” OR sarscov OR “sars virus” OR COVID OR Covid-19 OR pandemic<br><br>#5MeSH descriptor: [SARS-CoV-2] explode all trees
(#1 OR #2 OR #3) AND (#4 AND #5) New Articles from the Cochrane Library:	8 [Removed 7 protocols)

For peer review only

EMBASE

Retrieved April 14, 2022

#	Searches
1. Medical Abortion	('contraceptives, postcoital':ti,ab,kw OR 'medical abortion':ti,ab,kw OR 'medication abortion':ti,ab,kw OR 'abortifacient agents':ti,ab,kw OR 'mifepristone':ti,ab,kw OR mifegyne:ti,ab,kw OR mifégyne:ti,ab,kw OR mifeprex:ti,ab,kw OR mifepristone:ti,ab,kw OR amethopterin:ti,ab,kw OR cityl:ti,ab,kw OR cyprostol:ti,ab,kw OR cytolog:ti,ab,kw OR gastotec:ti,ab,kw OR gastrul:ti,ab,kw OR gymiso:ti,ab,kw OR hemoprostol:ti,ab,kw OR isprel:ti,ab,kw OR misel:ti,ab,kw OR misodel:ti,ab,kw OR misopress:ti,ab,kw OR misoprostil:ti,ab,kw OR misotrol:ti,ab,kw OR misoclear:ti,ab,kw OR misoprostol:ti,ab,kw)
2. Covid-19	'coronavirus disease 2019'
#1 AND #2 New Articles from EMBASE	71

For peer review only
